# BRD4 Short Isoform Interacts with RRP1B, SIPA1 and Components of the LINC Complex at the Inner Face of the Nuclear Membrane

**DOI:** 10.1371/journal.pone.0080746

**Published:** 2013-11-19

**Authors:** Jude Alsarraj, Farhoud Faraji, Thomas R. Geiger, Katherine R. Mattaini, Mia Williams, Josephine Wu, Ngoc-Han Ha, Tyler Merlino, Renard C. Walker, Allen D. Bosley, Zhen Xiao, Thorkell Andresson, Dominic Esposito, Nicholas Smithers, Dave Lugo, Rab Prinjha, Anup Day, Nigel P. S. Crawford, Keiko Ozato, Kevin Gardner, Kent W. Hunter

**Affiliations:** 1 Laboratory of Cancer Biology and Genetics, Center for Cancer Research, National Cancer Institute, National Institutes of Health, Bethesda, Maryland, United States of America; 2 Department of Biochemistry and Molecular Biology, School of Medicine, Saint Louis University, Saint Louis, Missouri, United States of America; 3 Laboratory of Proteomics and Analytical Technologies, Frederick National Laboratory for Cancer Research, Frederick, Maryland, United States of America; 4 Epinova DPU and Quantitative Pharmacology, Immuno-Inflammation Therapeutic Area, GlaxoSmithKline, Stevenage, United Kingdom; 5 Laboratory of Molecular Growth Regulation, National Institute of Child Health and Human Development, National Institutes of Health, Bethesda, Maryland, United States of America; 6 Cancer Genetics Branch, National Human Genome Research Institute, National Institutes of Health, Bethesda, Maryland, United States of America; 7 Genetic Branch, Center for Cancer Research, National Cancer Institute, National Institutes of Health, Bethesda, Maryland, United States of America; University of Alabama at Birmingham, United States of America

## Abstract

Recent studies suggest that BET inhibitors are effective anti-cancer therapeutics. Here we show that BET inhibitors are effective against murine primary mammary tumors, but not pulmonary metastases. *BRD4*, a target of BET inhibitors, encodes two isoforms with opposite effects on tumor progression. To gain insights into why BET inhibition was ineffective against metastases the pro-metastatic short isoform of BRD4 was characterized using mass spectrometry and cellular fractionation. Our data show that the pro-metastatic short isoform interacts with the LINC complex and the metastasis-associated proteins RRP1B and SIPA1 at the inner face of the nuclear membrane. Furthermore, histone binding arrays revealed that the short isoform has a broader acetylated histone binding pattern relative to the long isoform. These differential biochemical and nuclear localization properties revealed in our study provide novel insights into the opposing roles of BRD4 isoforms in metastatic breast cancer progression.

## Introduction

Breast cancer is the most common female malignancy in the United States [1]. It has been estimated that 90% of cancer-related mortality is due to metastatic disease rather than the primary tumor. Patients with disseminated tumors at diagnosis have universally poor outcomes relative to those with local disease. However, a significant proportion of patients with a localized tumor at diagnosis relapse with metastatic disease and these patients frequently succumb due to tumor-related events. The application of adjuvant therapy to target disseminated tumor cells after surgical resection of the primary lesion reduces, but does not eliminate, the incidence of subsequent metastatic disease in human breast cancer. A better understanding of the metastatic process and a focus on clinical targeting of metastatic disease is therefore critical to further reduce the morbidity and mortality of breast cancer.

Previously our laboratory identified the transcriptional elongation factor *Brd4* (Bromodomain-containing protein 4) as a metastasis susceptibility gene [2]. Using a genetically engineered mouse model of aggressive breast cancer we demonstrated that *Brd4* expression levels were associated with extracellular matrix genes, components of human breast cancer prognostic gene signatures. We subsequently demonstrated that increased expression of *Brd4* in orthotopic transplant models of mammary tumor significantly suppressed primary tumor growth and metastatic disease [2]. More recently we showed that the two isoforms of BRD4, which share the same N-terminal region except for the final three amino acids, have opposing roles in breast cancer growth and progression [3]. This is consistent with other previous hypothesis that the short isoform is a competitive inhibitor of the long isoform [3].

BRD4 has been of great interest due to the development of small molecule inhibitors of the bromodomains. The inhibitors have demonstrated efficacy against several tumor types [4-8], including the highly refractory midline carcinomas that result from BRD4-NUT translocation fusion [9,10]. These studies however, have not addressed the potential effect of the inhibitors against metastatic disease. Since BRD4 has both metastasis inhibiting and metastasis enhancing capacities due to the opposing effects of the two isoforms [2,3], the effect of these inhibitors on long term survival in breast cancer patients remains unclear.

Here we present data showing that while the small molecule bromodomain inhibitor I-BET151 [6] is effective at inhibiting primary tumor growth, it does not reduce pulmonary metastasis in two orthotopic models of metastasis. To gain further understanding on how bromodomain inhibitors can be refined to target metastatic tumors, we have further characterized the pro-metastatic short isoform of BRD4. We demonstrate that BRD4 short isoform (BRD4-SF) is a nuclear membrane-associated protein, while the long isoform (BRD4-LF) is associated with the nuclear matrix. BRD4-SF interacts with other metastasis susceptibility proteins such as SIPA1 (Signal-Induced Proliferation-Associated protein 1) and RRP1B (Ribosomal RNA Processing 1 homolog B). This complex interacts with the inner nuclear membrane protein SUN2 (Sad1 and UNC84 domain containing 2) at the inner face of the nuclear membrane and therefore may play a role in mechanotransduction of extracellular microenvironmental signals to the nucleus. Moreover, despite possessing identical bromodomains, BRD4-SF has an expanded histone binding affinity relative to BRD4-LF. These results shed light onto the mechanisms by which the two BRD4 isoforms differently regulate metastasis and provide a foundation for the refinement of bromodomain inhibitors as therapeutic agents in metastatic breast cancer and other solid tumors.

## Material and Methods

### Ethics Statement

All mouse experiments were carried out in strict accordance with the guidelines of the National Cancer Institute's Animal Care and Use Committee. All mouse experiments were approved by the National Cancer Institute's Animal Care and Use Committee. All mouse surgeries were performed under isoflurane anesthesia, and all efforts were made to minimize suffering.

### Cell Culture

HeLa cells [11] were obtained as a gift from Dr. Pei-Wen Chen from the laboratory of Dr. Paul Randazzo (National Cancer Institute, Bethesda, MD). For pull down experiments HEK293 cells were used at the Laboratory of Proteomics and Analytical Technologies, Frederick National Laboratory for Cancer Research. For other experiments HEK293 were obtained from ATCC. Both Mvt1 [12] and 6DT1 [12] cell lines were obtained as a gift from Dr. Lalage Wakefield (National Cancer Institute, Bethesda, MD) and were originally generated in Dr. Robert Dickson’s laboratory (Georgetown University Medical Center, Washington, D.C). All cell lines were maintained in Dulbecco’s modified Eagle’s medium supplemented with 10% fetal bovine serum, 2 mM glutamine, and 1% of penicillin/streptomycin and incubated at 37°C in 5% CO2.

### Animal Treatment with the I-BET151 Inhibitor

FVB/NJ female mice were injected at 6-week of age orthotopically into the forth mammary fat pad with 1x10^5^ cells per animal. Two cell lines were used in this study: Mvt1 or 6DT1 (20 mice for each cell line). After ten days of cell injection, which gives enough time for the cells to form primary tumors, 10 mice were used for treatment with I-BET151 and 10 mice were used for vehicle treatment. Mice were treated daily with intraperitoneal (ip) dosing at a target dose of 30 mg/kg of the I-BET151 compound, which was dissolved in 5:95 v/v DMSO, 10% w/v, Kleptose HPB in 0.9%/g saline, pH 5.0. Vehicle-treated mice were also given ip injection of 5:95 v/v DMSO, 10% w/v, Kleptose HPB in 0.9%/g saline daily. Twenty-seven days post cell injection (seventeen days post-treatment) primary tumor weight was compared, lungs were harvested, sectioned, H&E stained, and metastasis nodules counted. All mouse experiments were performed under animal study protocols approved by the National Cancer Institute's Animal Care and Use Committee.

### Expression Vectors

BRD4 vectors used were: BRD4-SF-FLAG expression vector contains a single FLAG epitope tag at the C-terminus of BRD4 short isoform. GFP-BRD4-SF is a BRD4-SF vector that contains a C-terminal fusion to eGFP. BRD4-SF-myc is a BRD4-SF lentiviral vector driven by mouse Pol2 promoter. This vector also contains a blasticidin resistant gene as a selection marker for stable expression. The vectors mentioned above were cloned into custom Gateway vectors by the Laboratory of Proteomics and Analytical Technologies, Frederick National Laboratory for Cancer Research. BRD4-LF-FLAG expression vector encodes a FLAG-tagged BRD4 long isoform [13]. BRD4-SF-Y433A-V5 and BRD4-LF-Y433A-V5 are vectors encoding BRD4-SF and BRD4-LF, respectively, with point mutations introduced in the second bromodomain of BRD4. Other vectors used were: mCherry-RRP1B vector was a gift from Dr. Laura Trinkle-Mulcahy [14]. RRP1B-HA vector was previously described [15]. mCherry-SIPA1, SIPA1-V5, SIPA1-myc, SIPA1+NLS, GFP-SUN2 vectors were cloned into custom Gateway vectors by the Laboratory of Proteomics and Analytical Technologies, Frederick National Laboratory for Cancer Research. The final expression clones were validated by restriction analysis and transfection-ready DNA was prepared using either the GenElute XP Maxiprep kit (Sigma) or the Endo-free Maxiprep kit (Qiagen). 

### Transfection, Affinity Purification of Protein Complexes and Electrophoresis

HEK293 cells were transfected and expressed proteins were purified as described [16]. Briefly, cells were lyzed with lysis buffer (50 mM Tris pH 7.4, 150 mM NaCl, 0.5 mM EDTA, 0.1% NP-40, and protease inhibitors cocktail), centrifuged and the supernatant was incubated with anti-FLAG M2 agarose (Sigma) for 2 hrs. The resin was then washed and the bait protein interactomes were eluted via a FLAG peptide challenge in elution buffer (6.25 mM NH_4_HCO_3_ pH 8.4, 150 μg/ml FLAG peptide) for 20 min, and the samples were lyophized. Samples were then incubated at room temperature (RT) for 5 min in 2x LDS-sample buffer (Invitrogen). The protein samples were then resolved on 4-12% Bis-Tris gels and each lane was cut into individual bands. Trypsin in 25 mM NH_4_HCO_3_ pH 8.4 was added to each sample and incubated on ice for 30 min. The resulting samples were incubated overnight at 37°C and the mixture of tryptic peptides was extracted. The combined extracts were lyophilized, reconstituted in 0.1% TFA and desalted. The eluate was lyophilized and reconstituted in 0.1% TFA and aliquots analyzed by nano RPLC-MS/MS as described below. 

### In-Solution Tryptic Digestion

The digestion was performed as described [16]. The lyophilized eluate obtained from the anti-FLAG M2 affinity purification was resuspended in 25 mM NH_4_HCO_3_ pH 8.4. The samples were digested overnight with trypsin at 37°C followed by lyophilization. The tryptic digest was reconstituted in 25% ACN/0.1% FA and fractionated using strong cation exchange (SCX) liquid chromatography (LC). 

### Nanoflow Reversed Phase Liquid Chromatography (NanoRPLC)-Tandem Mass Spectrometry (MS)

Nanoflow RPLC-MS/MS analyses were performed using an Agilent 1100 nanoflow LC system coupled online with a linear ion trap (LIT) mass spectrometer (LTQ from ThermoElectron) as described [16]. Briefly, microcapillary RPLC column was in-house slurry-packed with 5 µm, 300 Å pore size, Jupiter C-18 stationary phase (Phenomenex). After sample injection, the column was washed and peptides were eluted using a linear gradient. The LIT-MS was operated in a data dependent mode in which each full MS scan was followed by seven MS/MS scans wherein the seven most abundant molecular ions were dynamically selected for collision-induced dissociation using normalized collision energy of 35%.

### Nano RPLC-MS Data Analysis and Interpretation

Acquired MS/MS spectra were searched against a human protein database (March, 2009) using the SEQUEST algorithm implemented in BioWorks 3.2 package (ThermoElectron) using 1.5 Da for precursor ion tolerance and 0.5 Da for fragment ions tolerance with methionine oxidation included as dynamic modification. Only fully tryptic peptides with up to two miscleavages with charge state dependent cross correlation Xcorr ≥ 2.1 for [M+H]^1+^, ≥ 2.5 for [M+2H]^2+^ and ≥ 3.2 for [M+3H]^3+^ and delta correlation (Δ*C*_*n*_) ≥ 0.08 were considered as positive identification.

### Transfections and Cross-Linking Co-Immunoprecipitation (Co-IP) Assay

HEK293 cells seeded at a density of 2.5x10^6^ cells/10 cm plate were transfected with 3 µg of different expression vectors using X-tremeGENE 9 DNA transfection reagent as per the manufacturer’s protocol (Roche Applied Science). Twenty-four hours post-transfection, cross-linking Co-IP was performed according to [17]. Briefly, cell suspension of transfected cells was cross-linked with 2 mM DSP (DTSP; Dithiobis [succinimidyl propionate]; Lomant's Reagent) (ProteoChem) for 2 hrs at 4°C and cross-linking was terminated by addition of 20 mM Tris-HCl pH 8.0 for 15 min at RT. Proteins were then extracted in 1 ml Buffer C (10 mM HEPES pH 7.8, 150 mM NaCl, 10% Glycerol, 1% NP-40, 0.1% SDS, 0.5% Sodium deoxycholate, protease inhibitors, phosphatase inhibitors) and collected by centrifugation. Equal amount of protein was pre-cleared by rotation at 4°C using 60 μl of GammaBind G Sepharose beads (GE healthcare) and collected by centrifugation. Input samples were collected from the pre-cleared lysate. In a separate tube, 30 μl of the beads was incubated with the antibody in 1 ml of Buffer C on a rotator at 4°C for 2-3 hrs. The antibody-bound beads were collected via centrifugation and incubated with the pre-cleared extract overnight on a rotator at 4°C. The beads were recovered by centrifugation and washed six times with Buffer C. The immunoprecipitated proteins and the input samples were eluted with 25 µl of 4X SDS sample buffer containing reducing agent (Invitrogen) by incubating at 96°C. The input samples were also incubated at 96°C after adding SDS sample buffer containing reducing agent (Invitrogen). 

### Bimolecular fluorescence complementation (BiFC) Analysis

Gateway Entry clones with or without stop codons were used to generate BiFC mammalian expression clones using Gateway LR recombination (Life Technologies). Venus BiFC vectors were created by insertion of Venus fluorescent protein coding regions into a Gateway converted pcDNA3.1-zeo vector.  Inserted fragments consisted of the N-terminal portion of Venus (amino acids 1-157) or the C-terminal portion of Venus (amino acids 158-238) fragments. All vectors were converted to Gateway Destination vectors (Life Technologies) and subcloned by Gateway LR recombination using the manufacturer’s protocols (Life Technologies). Transfection-ready DNA was prepared using the GenElute XP Maxiprep kit (Sigma). To perform BiFC analysis [18,19], 1x10^5^ HeLa cells seeded into 2-well chamber slides (Thermo Scientific) were transfected with BiFC expression vectors using PolyFect transfection reagent as per the manufacturer’s protocol (Qiagen). Briefly, cells in every culture vessel were transfected with 1.2 µg of vector DNA and 10 µl of PolyFect transfection reagent. After 24 hrs the cells were incubated on a heat block at 30°C for 1 hr then fixed with 4% paraformaldehyde in PBS (USB Corporation) and permeabilized with 0.2% Triton X-100 (Sigma) in PBS (Lonza). Cells were stained with DAPI in PBS and the emission of YFP fluorescence was detected using a confocal microscope (Zeiss) at 63X magnification. 

### Immunofluorescence

HeLa cells seeded into 2-well chamber slides (Thermo Scientific) at a density of 1x10^5^ were transfected with different expression vectors using PolyFect transfection reagent as per the manufacture’s protocol (company). Twenty-four hours later cells were fixed with 4% paraformaldehyde in PBS (USB Corporation), permeabilized with 0.2% Triton X-100 (Sigma) in PBS (Lonza) and the emission of YFP fluorescence was detected using a confocal microscope (Zeiss) at 63X magnification. Pre-extraction was performed as described [20]. Briefly, 24 hrs post-transfection, HeLa cells were pre-extracted for 1 min using pre-extraction buffer (1% Triton X-100, 25 mM Tris-HCl pH 8.0, 150 mM KOAc, 15 mM NaCl, 5 mM MgCl2) then washed, fixed and processed as described above. 

### Cellular Fractionation-Lamond lab Protocol

The Lamond lab protocol was used as described in http://www.lamondlab.com/f7nucleolarprotocol.htm with few modifications. Briefly, ~90% confluent HeLa cells were pelleted and lyzed with hypotonic Buffer A (10 mM HEPES pH 7.9, 10 mM KCl, 1.5 mM MgCl2, 0.5 mM DTT, protease inhibitors) then homogenized 30 times using a Wheaton Dounce tissue homogenizer (Fisher Scientific). Cells were collected by centrifugation and the supernatant was kept as the cytoplasmic fraction. The pelleted cells were subjected to a sucrose gradient centrifugation followed by sonication and centrifugation. The upper supernatant was kept as the nuclear membrane-enriched fraction, the lower supernatant was kept as the nucleoplasmic fraction and the pellet was resuspended in S2 solution (0.35 M sucrose, 0.5 mM MgCl2) and kept as the nucleolar-enriched fraction.

### Cellular Fractionation-Gardner lab Protocol

The second protocol was obtained from Dr. Kevin Gardner’s lab as follows: ~90% confluent HeLa cells were pelleted and lyzed with hypotonic Buffer A (10 mM HEPES pH 7.5, 10 mM KCl, 1.5 mM MgCl_2_, 4 mM β-mercaptoethanol, protease inhibitors) then homogenized 32 times using a Wheaton Dounce tissue homogenizer (Fisher Scientific). Cells were collected by centrifugation and both supernatant and pellet (nuclear pellet) were kept. Supernatant was subjected to a high speed centrifugation resulting in the cytosolic fraction (the supernatant) and the pelleted membrane-enriched fraction was resuspended in PBS containing 0.5% Triton X-100. The nuclear pellet was resuspended in Buffer D (20 mM HEPES pH 7.5, 0.2 mM EDTA, 20% glycerol, 4 mM β-mercaptoethanol, protease inhibitors) and 4 M Ammonium Sulfate pH 7.9 was added for 30 min with gentle rocking then centrifuged at high speed. The supernatant was collected as the nuclear extract.

### Lentiviral Gene Transfer and Construction of Myc-Tagged BRD4-SF Cells

To construct cell lines that stably express myc-tagged BRD4-SF lentiviral particles were produced as described [3]. Briefly, HEK293 cells were transfected with BRD4-SF lentiviral vector, psPAX2 packaging plasmid, and pMD2.G envelope plasmid using X-tremeGENE 9 DNA transfection reagent as per the manufacturer’s protocol (Roche Applied Science). Virus-containing media was harvested, filtered and viral particles were then used to infect HeLa cells. Blasticidin was used as a selection marker for stable expression.

### Immunoprecipitation of the Nuclear Membrane-Enriched Fraction

Equal amount of nuclear membrane-enriched samples were pre-cleared by rotation at 4°C using 50 μl of GammaBind G Sepharose beads (GE healthcare) and collected by centrifugation. Input samples were collected from the pre-cleared lysate. In a separate tube, 40 μl of the beads was incubated with the antibody in 1 ml of RIPA buffer (50 mM HEPES, 20 mM sodium pyrophosphate, 25 mM β-glycerophosphate, 5 mM sodium molybdate, 5 mM EDTA, 150 μM Orthophenanthroline, 1% NP-40, 0.5% deoxycholate, 1% Triton X-100, protease inhibitors) on a rotator at 4°C for 2-3 hrs. The antibody-bound beads were collected via centrifugation and incubated with the pre-cleared extract overnight on a rotator at 4°C. The beads were recovered by centrifugation and washed with RIPA buffer. The immunoprecipitated proteins were eluted with 25 µl of 4X SDS sample buffer containing reducing agent (Invitrogen) by incubating at 96°C. The input samples were also incubated at 96°C after adding SDS sample buffer containing reducing agent (Invitrogen). 

### Formaldehyde Cross-Linking Immunoprecipitation (IP)

Formaldehyde cross-linking IP was performed according to [21]. HeLa cells ectopically expressing myc-tagged BRD4-SF were mildly cross-linked by addition of 1% formaldehyde directly to the culture media and incubated for 10 min at RT. The cross-linking reaction was stopped by adding 125 µM glycine and gentle shaking for 5 min. Cells were then scraped in PBS, pelleted, dissolved in ice-cold SDS lysis buffer (1% SDS, 10 mM EDTA, 50 mM Tris pH 8.1) and sonicated for 2X 30 sec at 10 μm amplitude using a tip sonicator. Equal amounts of the samples were pre-cleared by rotation at 4°C using 50 μl of GammaBind G Sepharose beads (GE healthcare) and collected by centrifugation. In a separate tube, 50 μl beads was incubated with anti-myc antibody in ChIP buffer [21] on a rotator at 4°C. The antibody-bound beads were collected via centrifugation and incubated with the pre-cleared extract overnight on a rotator at 4°C. The beads were recovered by centrifugation and washed with ChIP buffer. The immunoprecipitated proteins were eluted with 30 µl of 2X Laemmli sample buffer with β-mercaptoethanol (Sigma) by incubating at 96°C for 20 min.

### Western Blot Analysis

SDS-PAGE was performed using the XCell SureLockTM Mini-Cell (Invitrogen) with a NuPAGE Novex gels (Invitrogen). Proteins were then transferred to Immobilon-P membranes (Millipore), the membranes were blocked in Tris-buffered saline (TBS) containing 5% non-fat dry milk and 0.1% Tween-20 (TBST) and immunoblotted against different primary antibodies and their corresponding secondary antibodies. Immunoblots were visualized by enhanced chemiluminescence with an ECL plus Kit (GE Healthcare Life Sciences).

### Preparation of Cell Lysate and MODified Histone Peptide Array

HEK293 cells were transiently transfected with 3 µg of BRD4-LF-FLAG, BRD4-SF-FLAG or V5-tagged point mutants of BRD4-LF or BRD4-SF using X-tremeGENE 9 DNA transfection reagent as per the manufacturer’s protocol (Roche Applied Science). Twenty-four hours post-transfection cells were harvested and cell lysates were prepared using Golden Lysis Buffer (20 mM Tris pH 8.0, 400 mM NaCl, 5 mM EDTA, 1 mM EGTA, 1 mM sodium pyrophosphate, 10% Glycerol, 1% Triton X-100, protease inhibitors). Equal amount of proteins were used to perform the MODified Histone Peptide Array as per the manufacturer’s protocol (Active Motif) [22]. Briefly, the arrays were blocked by incubation in TTBS buffer (10 mM Tris HCl pH 7.5, 0.05% Tween-20, 150 mM NaCl) containing 5% non-fat dried milk at 4°C overnight. The arrays were then washed with interaction buffer (100 mM KCl, 20 mM HEPES pH 7.5, 1 mM EDTA, 0.1 mM DTT, 10% glycerol) and incubated with the lysate in interaction buffer for 2 hrs at RT. Arrays were washed with TTBS buffer and incubated with primary antibodies and the corresponding secondary antibodies, then submerged in ECL developing solution and visualized by enhanced chemiluminescence with an ECL plus Kit (GE Healthcare Life Sciences).

### Antibodies

Purchased primary antibodies were: anti-FLAG M2 (F3165, Sigma-Aldrich), anti-Myc-Tag (2276S, Cell Signaling Technology), high affinity anti-HA (11867423001, Roche Applied Science), anti-RRP1B (HPA017893, Sigma), anti-FBL (ab5821, Abcam), anti-LMNB1 (ab16048, Abcam), anti-α-Tubulin (T6199, Sigma), anti-SUN2 (HPA001209, Sigma), anti-NAT10 (GTX119166, GeneTex) and anti-SUN1 (GTX63537, GeneTex). Non-commercial BRD4 antibodies used were anti-N-terminal-BRD4 (BRD4-SF) and anti-C-terminal-BRD4 (BRD4-LF) [23]. 

Secondary antibodies used for immunofluorescence were: Alexa Fluor® 488 anti-mouse highly cross-adsorbed (A-11029, Invitrogen), Alexa Fluor® 594 anti-rabbit highly cross-adsorbed (A-11037, Invitrogen). 

### Statistical Analysis

Mann-Whitney test was used to calculate the *P* value for the I-BET151 animal study.

## Results

### I-BET151 Suppresses Primary Tumor Growth but Not Metastasis

**Figure 1:**
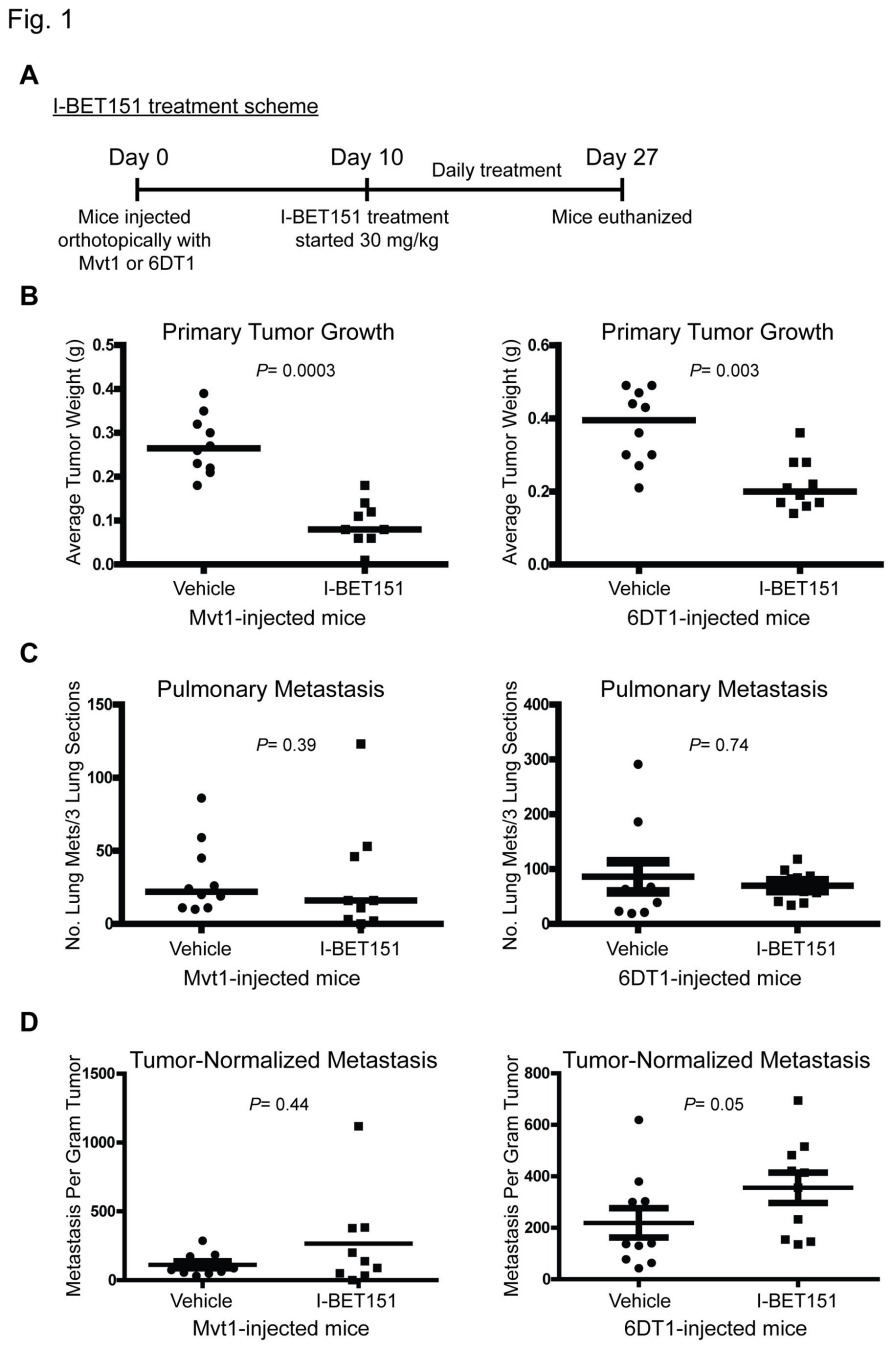
I-BET151 reduces primary tumor but not metastasis. (A) Layout of I-BET151 mouse experiment. FVB/NJ female mice were injected orthotopically into the mammary fat pad with Mvt1 or 6DT1 cells and 10 days post cell-injection, mice were treated daily with I-BET151 (30 mg/kg) or vehicle for 17 days. Mice were euthanized 27 days after cell injection. (B) Primary tumor weight of FVB/NJ female mice injected orthotopically into the mammary fat pad with Mvt1 or 6DT1 cell lines then treated with I-BET151 or vehicle. Weight assessed 27 days post-cell injection. (C) Quantification of pulmonary metastasis nodules after sectioning and H&E staining of lungs of mice injected orthotopically with cells then treated with I-BET151 or vehicle. (D) Normalization of pulmonary metastasis nodules count from H&E staining by primary tumor mass. Means ± SD, n=10 per group (n=9 for I-BET151-treated Mvt1-injected mice). *P* value calculated using Mann-Whitney test.

The small molecule inhibitor I-BET151 (GSK1210151A) has shown high efficacy for MLL fusion leukaemia models [6]. To test whether this compound has any effect on breast cancer metastasis, mice implanted with the highly metastatic mouse mammary tumor cell lines Mvt1 or 6DT1 [12] were treated with I-BET151 (30 mg/kg) or vehicle (Figure 1A) and primary tumor growth and lung metastasis were assessed. The first treatment with I-BET151 or vehicle was started 10 days post-cell injection to allow for the primary tumors to form before treatment. The experiment was terminated 27 days post-cell injection, a timeframe needed for metastasis to occur in our mouse model. As shown in Figure 1B, mice treated with I-BET151 had a reduced primary tumor weight compared to vehicle-treated mice. However, I-BET151 did not affect pulmonary metastasis from an orthotopic site (Figure 1C). To separate I-BET151 effect on tumor growth from its effect on metastasis and to allow for better comparison of metastatic capacity, metastasis counts were normalized by primary tumor burden, which confirmed that I-BET151 did not target metastasis (Figure 1D). 

Previously our laboratory demonstrated that the two BRD4 isoforms have opposite effects on tumor progression with BRD4-LF suppressing tumor growth and dissemination [2] and BRD4-SF promoting metastasis [3]. It is important to mention here that the two isoforms of BRD4 share the same N-terminal domain except for the final three residues, and with the short isoform lacking the C-terminal domain (Figure S1A). We, therefore, hypothesized that the lack of I-BET151 efficacy on metastatic disease is the result of greater inhibition of BRD4-LF compared to BRD4-SF, by differential drug accessibility or other isoform-specific biochemical properties. BRD4-LF is a well characterized transcriptional elongation factor while little is known about BRD4-SF. We initiated in-depth molecular and subcellular analysis of BRD4-SF to better understand its role in promoting metastasis and to identify unique features that could be targeted to disrupt metastatic progression.

### BRD4 Short Isoform Interacts with the Metastasis Susceptibility Protein RRP1B

To gain better understanding of the functional activities of BRD4-SF, candidate protein binding partners of BRD4-SF were identified by immunoprecipitation (IP)-mass spectrometry (MS) analysis [16] (Figure S1B). The Gene Ontology tool ToppGene Suite [24] revealed that BRD4-SF interacts with factors involved in a wide range of biological processes, including ribosome biogenesis, RNA processing, chromosome organization, chromatin modification and regulation of cell cycle, among many others (Figure S2). Detailed examination of the interaction data revealed the presence of a number of nucleolar proteins, one of which is the metastasis susceptibility protein RRP1B (Table S1). Previously described RRP1B interacting proteins [15] were also observed in BRD4-SF peptide list (Table S2). Due to the known roles of RRP1B in breast cancer progression and metastasis, further efforts were focused on validating its interaction with BRD4-SF.

**Figure 2:**
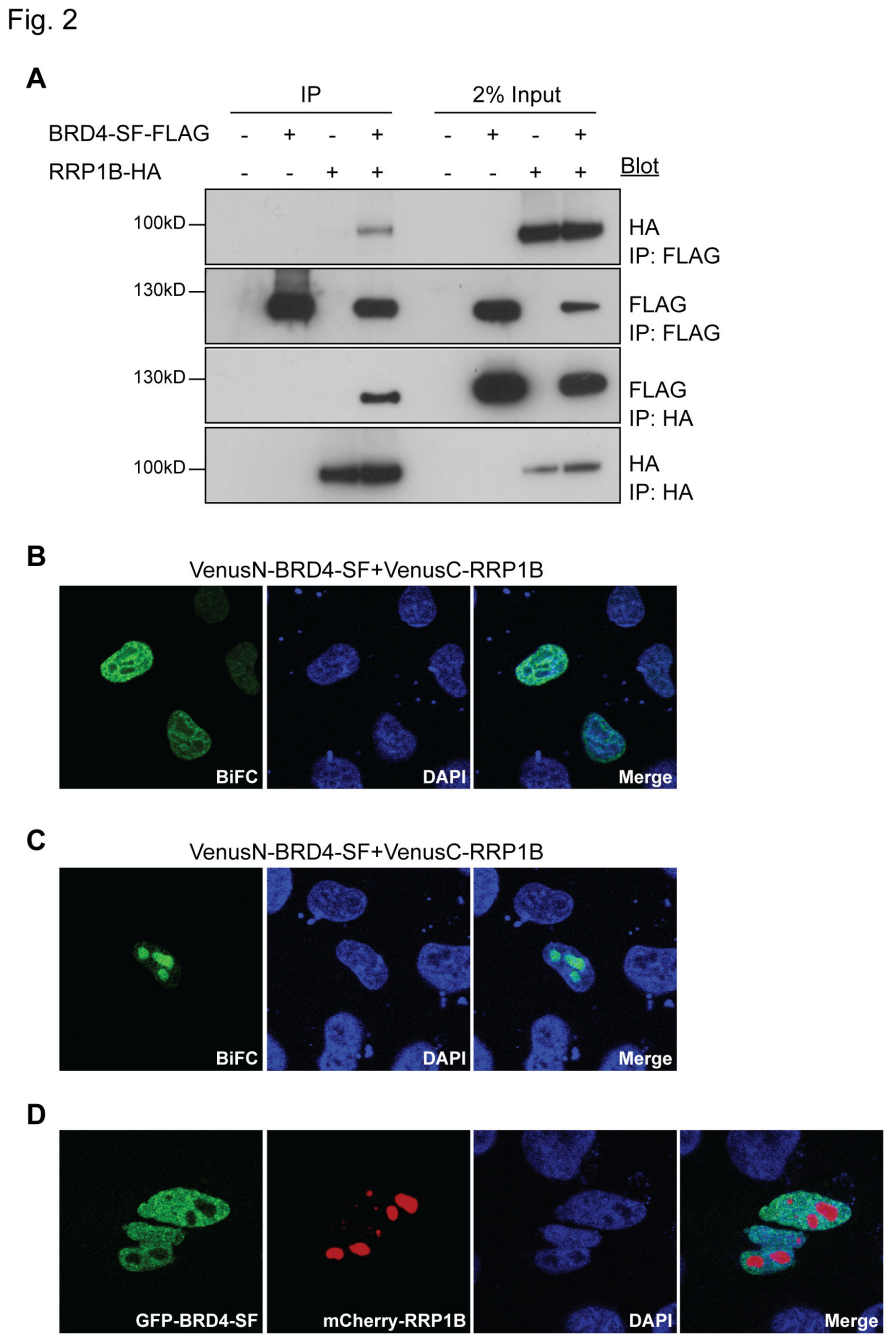
BRD4-SF interacts with RRP1B. (A) Interaction of epitope-tagged BRD4-SF and RRP1B by reciprocal Co-IP in HEK293 cells using antibodies against the epitope tags, followed by western blot analysis. (B, C) BiFC analysis of BRD4-SF and RRP1B in HeLa cells followed by confocal microscopy. Green: fluorescence complementation, blue; DAPI: double-stranded DNA. (D) Co-immunofluorescence of GFP-BRD4-SF (green) and mCherry-RRP1B (red) in HeLa cells followed by confocal microscopy. Blue; DAPI: double-stranded DNA. All pictures were taken at 63x magnification using confocal microscopy.

Since BRD4-SF is identical to the amino-terminal half of BRD4-LF, except for the final three amino acids, no BRD4-SF-specific antibody is available. Reciprocal co-immunoprecipitation (Co-IP) using epitope-tagged BRD4-SF and RRP1B was therefore performed. As shown in Figure 2A, reciprocal Co-IP revealed an interaction between the two molecules. Further, bimolecular fluorescence complementation (BiFC) assay between BRD4-SF and RRP1B was performed to confirm the interaction and to determine the localization of the potential BRD4-SF/RRP1B complex. BiFC analysis demonstrated two patterns of localization: diffusely throughout the nucleus (Figure 2B), or concentrated within the nucleolus (Figure 2C). Although RRP1B is primarily thought to be nucleolar [14,25] it has been detected in other nuclear compartments [15]. These results are therefore consistent with BRD4-SF and RRP1B interaction and suggest that dynamic relocalization of the interaction may occur within the nucleus.

### BRD4 Isoforms Localize to Distinct Subnuclear Compartments

The interaction between BRD4-SF and RRP1B in the nucleolus was unexpected and co-transfection of GFP-BRD4-SF and mCherry-RRP1B did not suggest nucleolar co-localization (Figure 2D). These results raised the possibility that the observed interaction between BRD4-SF and RRP1B was a result of relatively rare interactions below the detection threshold of fluorescence detection or due to experimental transfection-induced mis-localizations.

**Figure 3:**
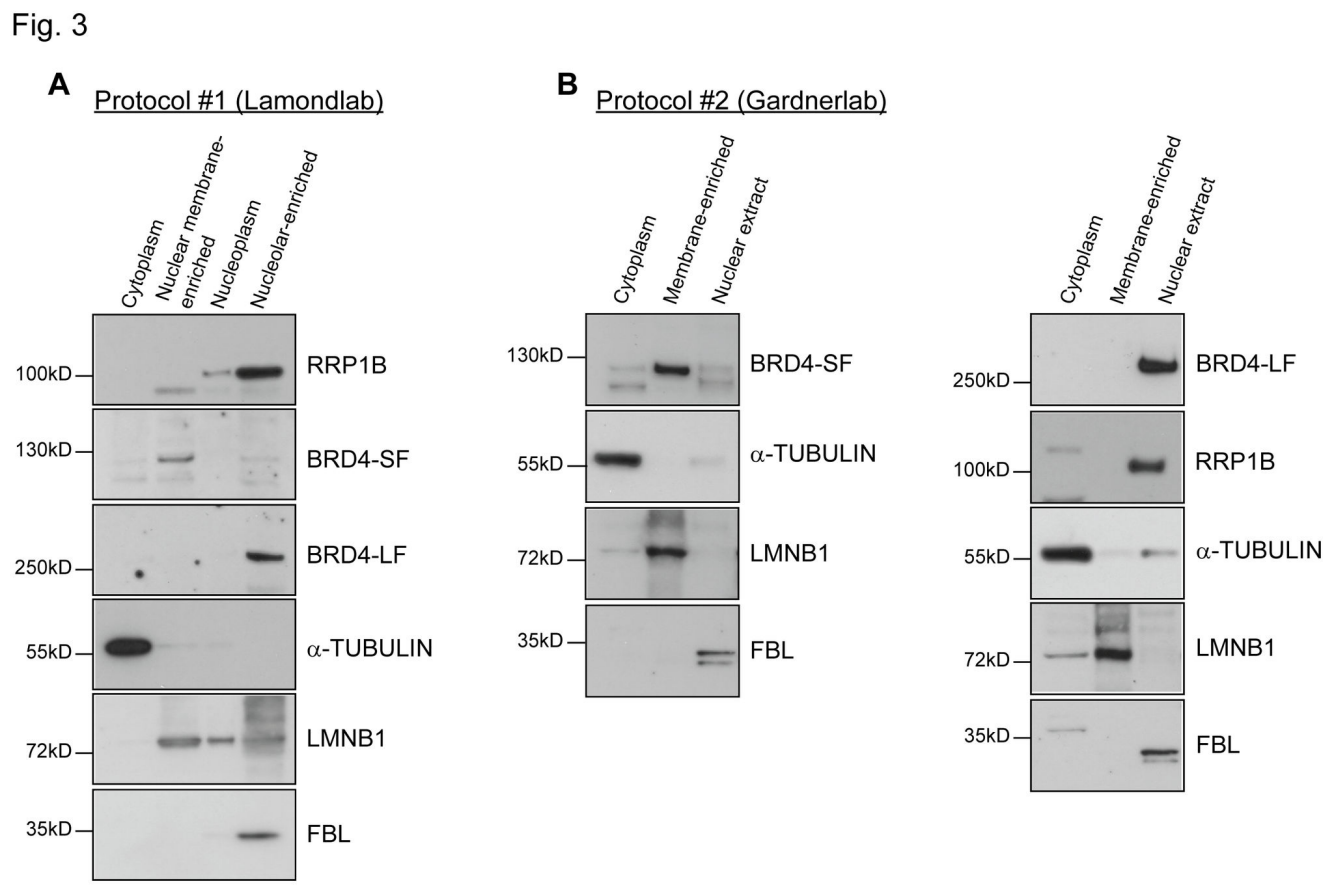
BRD4-SF localizes at the nuclear membrane. (A, B) Cellular fractionation experiments of HeLa cells using either Lamond lab protocol, protocol 1 (A) or Gardner lab protocol, protocol 2 (B) followed by western blot analysis.

To address this possibility, subcellular fractionation was performed to examine the subnuclear localization of BRD4-SF. Nuclei were isolated from untransfected HeLa cells, disrupted by sonication, and the large particulate nuclear bodies, including the nucleolus and transcriptionally active nuclear matrix, were pelleted by low speed centrifugation as described by the Lamond lab [26] (Figure S3). Western blot analysis of the subcellular fractions revealed that RRP1B was mainly present in the nucleolar-enriched fibrillarin (FBL)-containing pellet (Figure 3A). An antibody against an epitope between the two bromodomains of BRD4 [23] revealed that the majority of BRD4-SF was retained in the fraction containing nuclear envelope lipids and higher levels of lamin B1 (LMNB1) (Figure 3A). Unexpectedly, BRD4-LF was uniquely localized in the nucleolar-enriched pellet (Figure 3A). These data suggest that despite BRD4-SF having the identical N-terminal sequence except for the terminal three residues the two isoforms reside in physically different subnuclear compartments.

A second, independent cellular fractionation protocol (Figure S4) confirmed that the majority of BRD4-SF was found in the membrane-enriched pellet generated after high-speed centrifugation, indicative of an association with the nuclear membrane (Figure 3B, left panel). Moreover, as with the sonication protocol, BRD4-LF was found uniquely in the salt extractable nuclear fraction (Figure 3B, right panel), consistent with the notion that the two isoforms localize to separate subnuclear regions. Interesting, RRP1B was also uniquely found in the salt extractable nuclear fraction (Figure 3B, right panel), suggesting that RRP1B resides within different locations of the nucleus.

### BRD4-SF Interacts with NAT10 and the LINC Complex Proteins SUN1 and SUN2

**Figure 4:**
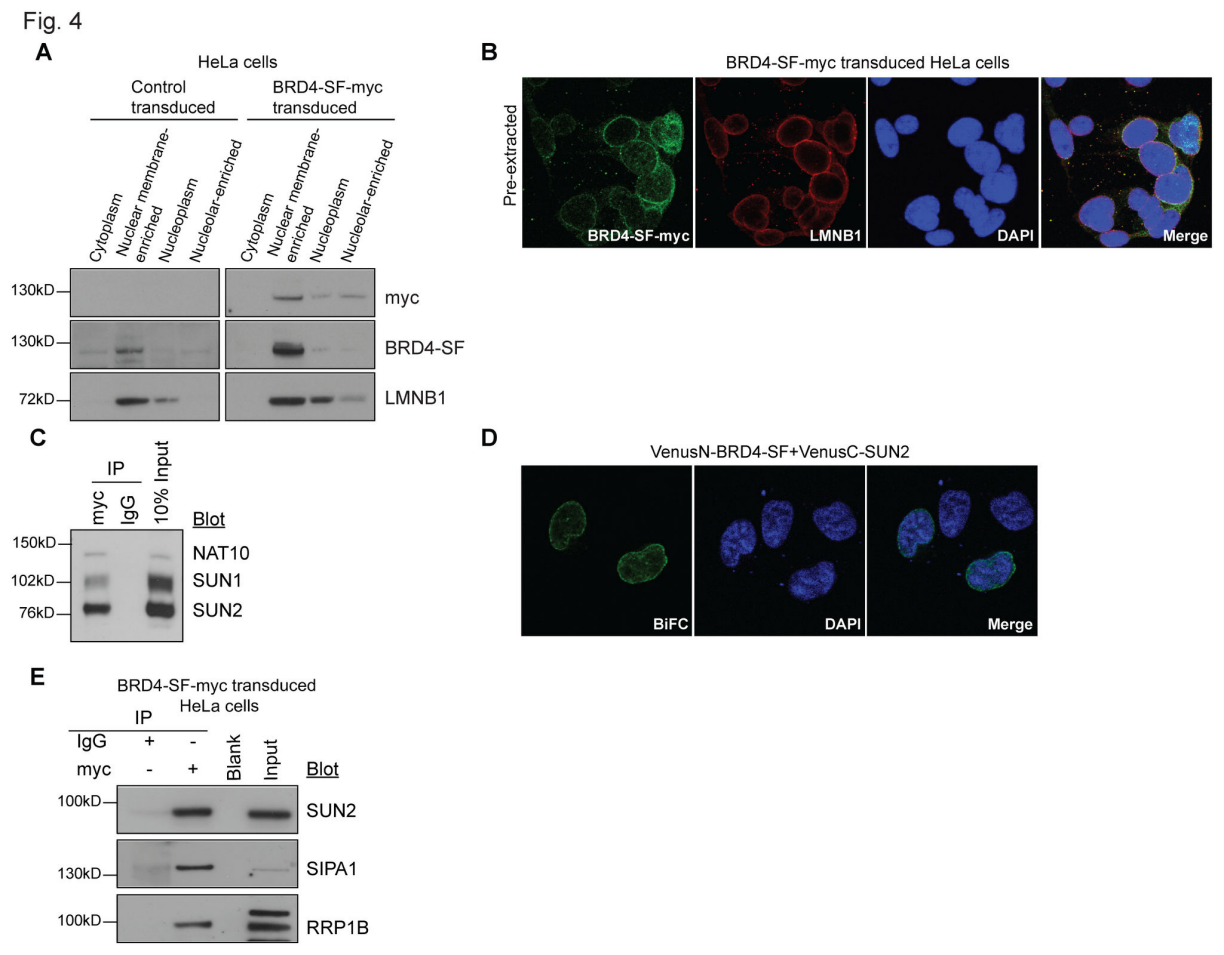
Epitope-tagged BRD4-SF localizes at nuclear membrane and interacts with SUN2. (A) Cellular fractionation of HeLa cells stably expressing myc-tagged BRD4-SF using Lamond lab protocol followed by western blot analysis. Control cells were transduced with an empty vector. (B) Confocal microscopy showing co-immunofluorescence of HeLa cells ectopically expressing myc-tagged BRD4-SF (green) after co-staining with the nuclear envelope marker lamin B1 (LMNB1; red) and double-stranded DNA (DAPI; blue). (C) Immunoprecipitation of HeLa cells stably expressing myc-tagged BRD4-SF using modified formaldehyde cross-linking protocol and anti-myc antibody for immunoprecipitation followed by western blot analysis. (D) BiFC analysis of BRD4-SF and SUN2 in HeLa cells followed by confocal microscopy. Green: fluorescence complementation, blue; DAPI: double-stranded DNA. (E) Immunoprecipitation from nuclear membrane-enriched fraction of HeLa cells ectopically expressing myc-tagged BRD4-SF using anti-myc antibody for immunoprecipitation followed by western blot analysis. All pictures were taken at 63x magnification using confocal microscopy.

The nuclear membrane association of BRD4-SF was assessed by both cell biology and biochemical methods. To specifically detect BRD4-SF, stable epitope-tagged expressing cells were generated. Myc-tagged BRD4-SF lentivirus was constructed, expressing the transcript from the low efficiency Pol2 promoter. HeLa cells were infected at a low multiplicity of infection, selected for integration and propagated under selection (Figure S5). The heterogeneous transduced population was harvested and cellular fractionation performed. Western blot analysis revealed that epitope-tagged BRD4-SF was expressed ~3-5x above endogenous levels, and was primarily localized in the nuclear membrane-enriched fraction (Figure 4A). Further, the nuclear envelope localization of myc-tagged BRD4-SF was examined by immunofluorescence (IF). Overexpression of nuclear envelope proteins tends to saturate binding sites at the nuclear envelope and accumulate in the endoplasmic reticulum, making clear confirmation of nuclear envelope targeting difficult. As a result, pre-fixation detergent extraction was used to remove this extraneous material [20]. As shown in Figure 4B, myc-tagged BRD4-SF localizes within the nuclear envelope and co-localizes with LMNB1, confirming nuclear envelope localization. 

To evaluate the nuclear envelope association of BRD4-SF, MS-derived candidate interaction partners were re-examined. The MS peptide list was compared to previous work that identified nuclear envelope-associated proteins [20]. Interestingly, BRD4-SF interacted with a large number of nuclear envelope proteins (Table S1). The histone acetyltransferase protein NAT10 was identified as the top inner nuclear membrane protein and was selected for Co-IP validation. Due to the challenge of identifying protein interactions in the nuclear lamina [27], a modified formaldehyde cross-linking protocol was performed [21]. As predicted, IP of myc-tagged BRD4-SF specifically pulled down endogenous NAT10 (Figure 4C).

 Previous studies have demonstrated a specific association of NAT10 with the inner nuclear transmembrane protein SUN1, a member of the LINC (linkers of the nucleoskeleton to the cytoskeleton) complex that connects the nuclear lamina to the cytoskeleton [28]. To further validate the association of BRD4-SF with the inner face of the nuclear membrane IP for SUN1 and the related inner nuclear transmembrane protein SUN2 was performed. IP with BRD4-SF-myc revealed specific interaction with both endogenous SUN1 and SUN2 (Figure 4C), confirming localization of BRD4-SF to the inner face of the nuclear membrane.

Due to yeast two hybrid data suggesting an interaction with SIPA1, the inner nuclear transmembrane protein SUN2 has been of interest in our laboratory. To determine if BRD4-SF was in proximity to SUN2 BiFC analysis was performed. BRD4-SF productively reconstituted the Venus-fluorophore when the SUN2-tag was nucleoplasmic (Figure 4D). As a control for potential mis-localization, BiFC analysis was also performed for SUN2 and BRD4-LF. No fluorescence complementation was detected (data not shown), consistent with the absence of BRD4-LF at the nuclear membrane. IP experiment with anti-myc was then performed on the nuclear membrane-enriched fraction from the Lamond lab protocol, and blotted for endogenous SUN2. As shown in Figure 4E, endogenous SUN2 interacted with BRD4-SF, confirming the interaction between these two molecules and suggesting a biologically relevant complex at the inner face of the nuclear membrane.

### BRD4-SF Interacts with the Metastasis Susceptibility Protein SIPA1 at the Nuclear Membrane

**Figure 5:**
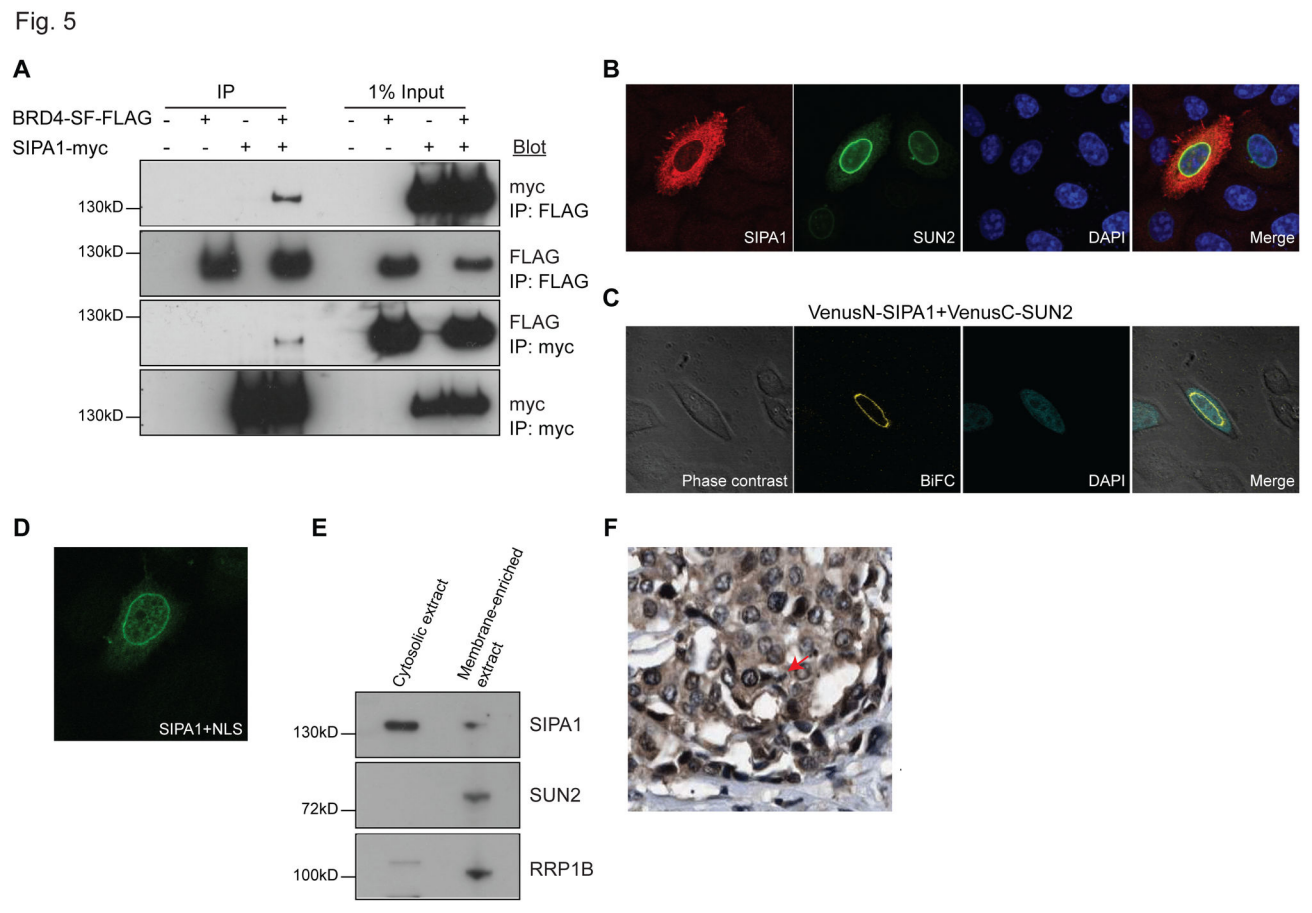
BRD4-SF interacts with SIPA1. (A) Interaction of epitope-tagged BRD4-SF and SIPA1 by reciprocal Co-IP in HEK293 cells using antibodies against the epitope tags, followed by western blot analysis. (B) Co-immunofluorescence of epitope-tagged SIPA1 (red) and SUN2 (green) in HeLa cells. Blue; DAPI: double-stranded DNA. (C) BiFC analysis of SIPA1 and SUN2 in HeLa cells followed by confocal microscopy. Green: fluorescence complementation, blue; DAPI: double-stranded DNA. (D) Immunofluorescence of a SIPA1 construct that has an SV40 nuclear localization signal (green) in HeLa cells followed by confocal microscopy. (E) Western blot analysis performed on the cytoplasmic fraction of HeLa cells isolated using Lamond lab cellular fractionation protocol after high speed centrifugation at 100,000 x g. Supernatant: cytosolic extract, pellet: membrane-enriched extract. (F) Immunohistochemical staining of breast cancer TMAs from the Human Protein Atlas for SIPA1 protein. All confocal microscopy pictures were taken at 63x magnification.

Previous studies demonstrated that the N-terminal region of SIPA1 encompassing the GAP domain interacts with the bromodomain II region of BRD4-LF [29]. Further, SIPA1 has been shown to interact with RRP1B [30], suggesting the three proteins form a complex. BRD4-SF and RRP1B interaction raised the possibility that BRD4-SF might also interact with SIPA1. To address this, reciprocal Co-IP between epitope-tagged SIPA1 and BRD4-SF was conducted and showed interaction between these two molecules (Figure 5A). 

The interactions of SIPA1 with both BRD4 isoforms and RRP1B are consistent with a nuclear localization. However, although SIPA1 has been described as a nuclear component, most reports suggest a cytoplasmic localization [31-33]. To resolve this discrepancy, subcellular localization studies of SIPA1 were performed. IF of SIPA1 revealed predominant cytoplasmic localization (Figure 5B). In a subset of cells (<5%) however, accumulation of SIPA1 was observed at the nuclear periphery. Co-staining with SUN2 suggested an accumulation of SIPA1 at the inner face of the nuclear membrane (Figure 5B), consistent with yeast two hybrid analysis (data not shown), suggesting an association between these two proteins. As shown in Figure 5C, SIPA1 and SUN2 BiFC analysis resulted in fluorescence complementation only for SUN2 constructs carrying the Venus-tag on the nucleoplasmic N-terminus of SUN2. No complementation was observed for constructs bearing the Venus-tag on the C-terminus which resides in the perinuclear space (data not shown). 

To further examine the nuclear distribution of SIPA1, the proportion of cells with high nuclear SIPA1 was enhanced by utilizing an SV40 nuclear localization signal-tagged SIPA1 construct. Consistent with BiFC and IF results, nuclear SIPA1 was concentrated at the nuclear periphery (Figure 5D). Finally, the localization of endogenous SIPA1 was examined by nuclear fractionations and human breast cancer tissue microarrays (TMAs) available through the Human Protein Atlas [34]. High speed centrifugation was performed on the cytoplasmic extract of the Lamond lab cellular fraction protocol to concentrate the membrane-containing fraction. Although the majority of endogenous SIPA1 was found to be cytoplasmic, endogenous SIPA1 was also observed in the membrane-concentrated fraction (Figure 5E). Immunohistochemical staining of the breast cancer TMAs from the Human Protein Atlas also showed predominantly cytoplasmic SIPA1, however staining at the nuclear periphery was observed in many cancer cells (Figure 5F). IP of BRD4-SF from the nuclear membrane-containing cell fractions also specifically pulled down endogenous SIPA1 (Figure 4E). In summary, BRD4-SF and SIPA1 interact, near or at the inner face of the nuclear membrane, suggesting that SIPA1 may dynamically shuttle across the nuclear membrane. Further, the localization results indicate that SIPA1 interacts predominantly with BRD4-SF, rather than BRD4-LF, since the molecules are physically separated within the nucleus. 

### BRD4-SF Interacts with RRP1B at the Nuclear Periphery

**Figure 6:**
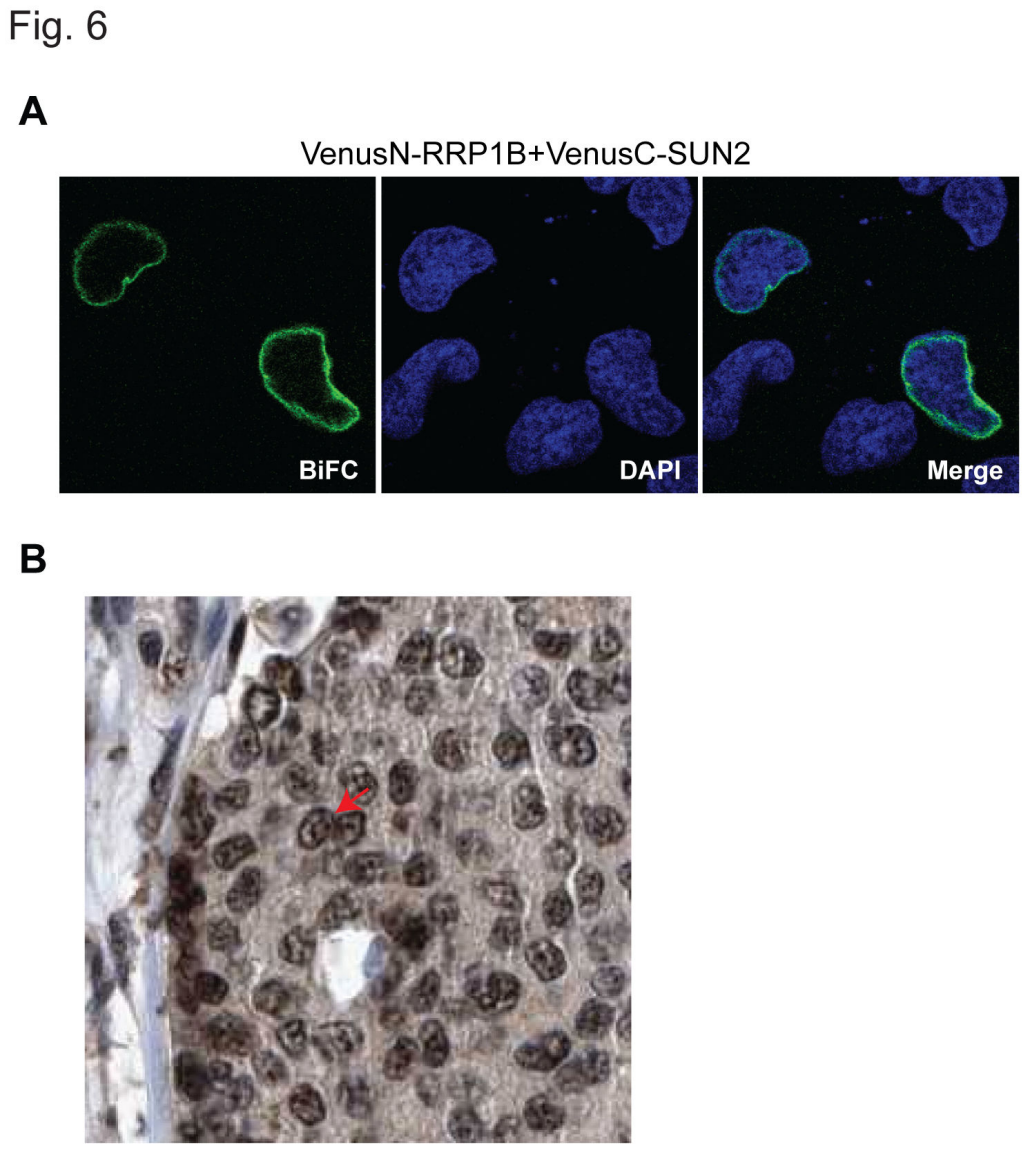
BRD4-SF interacts with RRP1B at the nuclear periphery. (A) BiFC analysis of RRP1B and SUN2 in HeLa cells followed by confocal microscopy. Green: fluorescence complementation, blue; DAPI: double-stranded DNA. Pictures were taken at 63x magnification using confocal microscopy. (B) Immunohistochemical staining of breast cancer TMAs from the Human Protein Atlas for RRP1B expression.

RRP1B, like SIPA1, has been reported in multiple cellular locations, primarily as a nucleolar protein and also a nucleolar and nuclear membrane-associated protein [14,15,25]. To test whether the above Co-IP between RRP1B and BRD4-SF was physiologically relevant, further characterization was performed. BiFC analysis between RRP1B and SUN2 demonstrated that exogenously expressed RRP1B was present at the nuclear periphery, as predicted (Figure 6A). Immunohistochemical staining of TMAs from the Human Protein Altas [34] revealed staining at both nucleolar and nuclear envelope locations in human breast cancer patient samples (Figure 6B). Endogenous RRP1B was also observed in the membrane-enriched fraction after high speed centrifugation (Figure 5E). Finally, IP of myc-tagged BRD4-SF from the nuclear membrane-enriched fraction specifically precipitated endogenous RRP1B (Figure 4E). Taken together these data strongly suggest that RRP1B resides within the nuclear lamina in addition to the nucleolus.

### BRD4-SF Has an Expanded Histone Binding Range Compared to BRD4-LF

Relative to BRD4-LF, BRD4-SF has the identical N-terminal sequence for all but the final few amino acids and lacks the C-terminal domain. Differential localization of the two isoforms may therefore be the result of: exclusion of BRD4-LF by physical occlusion from the nuclear lamina, masking protein-protein interaction sites due to the bulky proline-rich C-terminus, or differential binding of the two isoforms due to differences in conformation of the bromodomains. Previous studies have demonstrated binding of BRD4 bromodomains to acetylated histones [35-38]. However, this data was generated with isolated recombinant bromodomains, rather than full length proteins, which might have unanticipated constraints not observed in the recombinant fragments.

**Figure 7:**
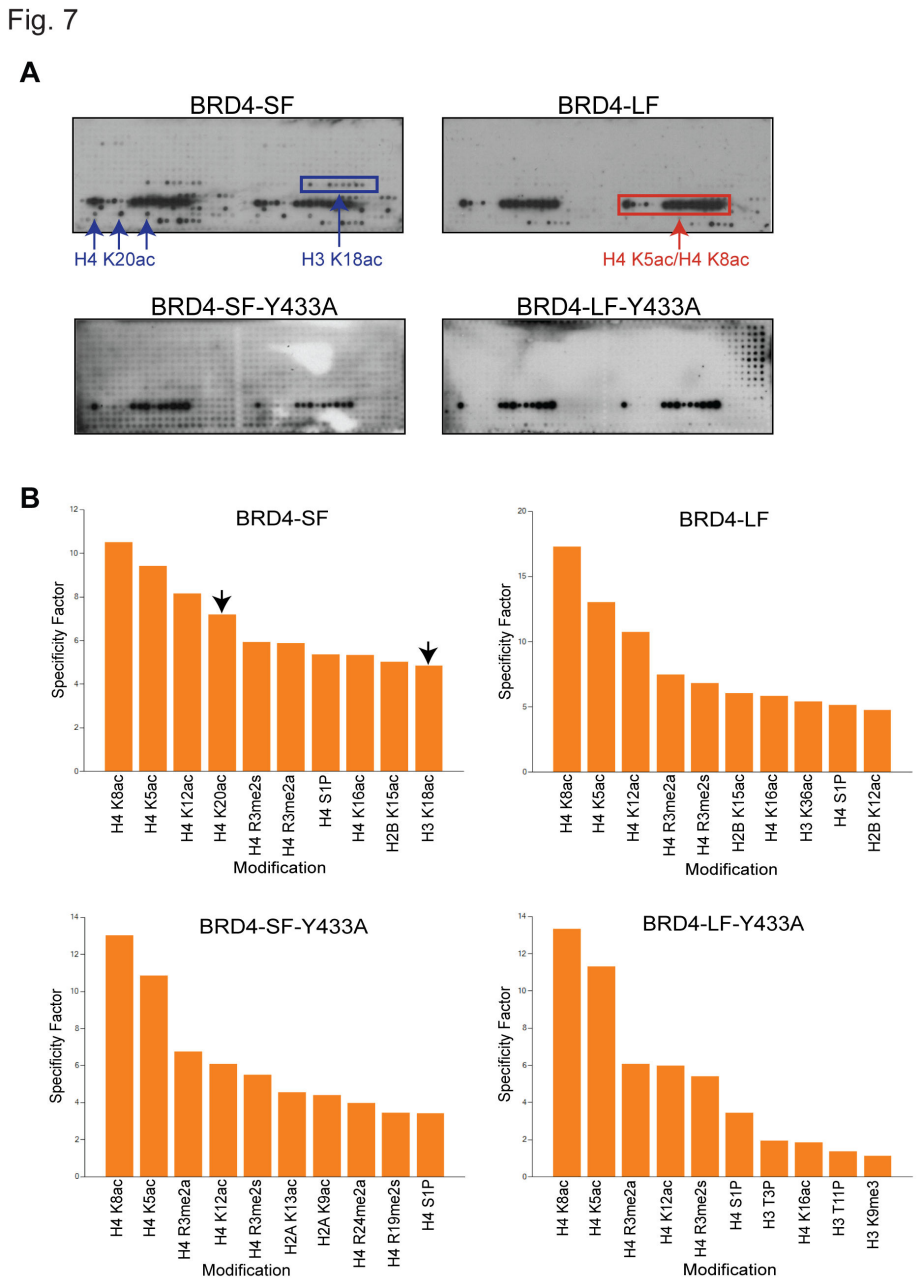
BRD4 isoforms have different binding affinities to histones. (A) MODified Histone Peptide Array of whole cell lysate from HEK293 cells transiently transfected with FLAG-tagged BRD4-LF, FLAG-tagged BRD4-SF or V5-tagged point mutations of both isoforms. The arrays were visualized using ECL plus western blot detecting reagent. (B) The specificity factor calculated as the ratio of average intensity of all spots containing the mark divided by average intensity of all spots not containing the mark. The graph shows the 10 modifications with the greatest specificity factors.

To explore this possibility, whole cell lysates from cells transiently transfected with epitope-tagged BRD4-SF or BRD4-LF were used to screen the Active Motif MODified Histone Peptide Array [22]. BRD4-LF strongly bound diacetylated H4K5/K8 peptides (Figure 7A and B, upper right panels). In contrast, BRD4-SF not only bound diacetylated H4K5/K8, but also strongly bound the plant specific acetylated H4K20, as well as acetylated H3K18 and H3K14/K18 diacetylated peptides (Figure 7A and B, upper left panels). Neither isoform bound acetylated H3K9, as previously reported for recombinant bromodomains [37].

Binding of acetylated H3K14 and H3K18 has been primarily associated with bromodomain II [37], suggesting that BRD4-SF’s expanded histone repertoire is due to an unmasking or a conformation change of bromodomain II compared to BRD4-LF. To address this question, point mutations [35,36] were introduced into epitope-tagged constructs of both isoforms to disrupt binding of the individual bromodomains. Y139A substitutions in bromodomain I did not significantly alter binding in either isoform compared to wild-type constructs (data not shown). In contrast, introduction of Y433A into bromodomain II did not alter BRD4-LF binding but eliminated all but the H4K5/K8 diacetylated peptide binding in BRD4-SF (Figure 7A and B, lower panels). As expected, a double point mutation (Y139A/Y433A) completely eliminated histone binding for both isoforms (data not shown). Since the Y139A mutant of BRD4-LF is capable of binding diacetylated H4K5/K8, these results are consistent with greater accessibility or a conformational change of bromodomain II in BRD4-SF that permits binding to acetylated H3K14/K18 peptides.

## Discussion

The discovery and optimization of small molecule inhibitors of epigenetic targets is a major focus of current biomedical research [39]. Small molecule inhibitors of bromdomain BET family of chromatin adaptor proteins have shown promising therapeutic effect in acute myeloid leukemia, multiple myeloma and Burkitt’s lymphoma [4-7]. These inhibitors have also shown promising results for an incurable, genetically-defined NUT-midline human squamous carcinoma [9,10], defined by the presence of acquired chromosomal rearrangements involving *BRD4-NUT* fusion genes. 

BRD4, a BET inhibitor target, has two alternatively spliced isoforms that exhibit opposite effects on metastasis. We previously showed that BRD4 long isoform (BRD4-LF) reduces metastasis [2], while the short isoform (BRD4-SF) promotes metastasis [3]. In the current study the small molecule inhibitor I-BET151 showed efficacy against the primary tumor in our breast cancer metastasis model, consistent with the previous tumor efficacy studies. Unexpectedly however, I-BET151 exhibited no efficacy against pulmonary metastasis. These findings were surprising since I-BET151 is expected to target both isoforms, which contain identical chromatin binding bromodomains. Furthermore, the BET inhibitors have shown to target c-Myc amplified tumors [5-8], however inhibition of MYC in human tumors might at times be contraindicated because its suppression may promote metastasis [40].

Our results suggest several plausible explanations. First, it is possible that the two isoforms are not inhibited equally by the inhibitor. If the inhibitor is more active against BRD4-LF the result may be equivalent to up-regulation of BRD4-SF, which we have previously shown to increase pulmonary metastatic colonization [3]. Differential activity against the two isoforms could be due to differences in post-translational modifications and/or conformational differences that alter bromodomain accessibility or structure. The histone binding assays presented here are consistent with this possibility, with BRD4-SF having a greater range of acetylated histone binding than BRD4-LF, mediated by bromodomain II. It is of interest that all of the modified histone binding of the bromodomains observed has been previously described using isolated bromodomain constructs. The specificity of the different isoforms however was only observed using full length proteins, suggesting that additional conformational and/or post-translational factors contribute to chromatin binding. Intriguingly, a recent study supports the potential role for casein kinase II phosphorylation induced conformational changes in BRD4-LF that alters bromodomain II accessibility [41]. Further investigation will be required to determine whether this mechanism is responsible for the differential binding of the two BRD4 isoforms to chromatin.

To gain better understanding of the pro-metastatic functions of BRD4 we opted to investigate the functional activities of the metastasis promoting short isoform. Although BRD4-LF has been extensively studied, little is known about BRD4-SF. Detailed characterization of the unique biology of BRD4-SF will lead to improved inhibitors and strategies that are effective against both the primary and metastatic tumors. 

One of the strategies we pursued was to perform protein-protein interaction experiments of BRD4-SF to identify unique interactions to this isoform. Unexpectedly the analysis showed that BRD4-SF interacts with a diverse range of proteins, many of which are nucleolar, including RRP1B, a previously described metastasis susceptibility protein [15,30]. Although BRD4 is known to have a Nuclear Localization Signal (NLS) within the N-terminal region [42], the localization of BRD4-SF has not been previously reported and therefore, we performed fluorescent epitope-tagged transfections to study cellular localization. Two patterns were observed; intense staining of the nucleoli, consistent with the mass spectrometry data, or diffuse staining throughout the nucleus, excluding the nucleolar regions. RRP1B has been described as a nucleolar protein [14,25] or both a nuclear envelope and nucleolar protein [15]. The BRD4-SF results imply dynamic relocalization within the nucleus. However, concerns about overexpression strategies led us to perform subnuclear fractionation to confirm endogenous protein localization.

Surprisingly, nuclear fractionation demonstrated that BRD4-SF does not localize within the nucleolus but at the nuclear envelope, while BRD4-LF is exclusively associated with the transcriptionally active nuclear matrix. The localization of BRD4-SF at the nuclear envelope was further supported by the presence of a large number of previously identified inner nuclear envelope-associated proteins [20] in our MS interaction data. Examination of the published nuclear envelope MS data [20] suggested that RRP1B, SIPA1 and many of SIPA1-associated yeast two hybrid targets [15] were also associated with the inner nuclear membrane. 

We have previously shown that a relationship between BRD4-LF, RRP1B and SIPA1 is critical for tumor progression and extracellular matrix regulation [43]. SIPA1 and RRP1B are known polymorphic metastasis susceptibility genes both in mice [30,44] and humans [30,45,46]. SIPA1 physically interacts with BRD4-LF [29] and RRP1B [30]. Further, SIPA1 has been predicted to interact with the inner nuclear membrane protein SUN2 by yeast two hybrid (unpublished data), which is part of the LINC (linkers of the nucleoskeleton to the cytoskeleton) complex connecting the interior of the nucleus to cytoskeleton. Taken together these data raised the possibility that these three metastasis susceptibility proteins form a complex on the inner face of the nuclear membrane. Bimolecular fluorescence complementation and immunoprecipitation of nuclear envelope-enriched cellular fraction from BRD-SF-transduced cell culture were consistent with an interaction between BRD4-SF and endogenous RRP1B, SUN2 and SIPA1 at the nuclear membrane. No evidence of interaction between BRD4-LF and SUN2 was observed, consistent with different subnuclear localizations. 

The nuclear envelope localization of BRD4-SF in complex with SUN2, SIPA1 and RRP1B raises a number of interesting possibilities. First, while the GFP-tagged BRD4-SF results suggest that many of the putative nucleolar protein interactions may not exist, a number of these proteins are also found in association with the nuclear membrane (ex. NAT10). Recent studies demonstrated that nucleolar-associated chromatin domains are capable of associating with the nucleolus or the nuclear membrane, and can reposition themselves after cell division [47]. In addition, nucleolar-associated domains have been shown to significantly overlap lamina-associated domains and have been suggested to alternate between the nucleolar and nuclear periphery [47]. Thus it is possible that both the nuclear envelope and nucleolar protein interactions are biologically relevant. BRD4-SF may therefore be associated with dynamic chromatin architecture reorganization that is dependent on cellular differentiation and state.

Second, the interaction of BRD4-SF, RRP1B and SIPA1 with the LINC complex suggests a role for mechanotransduction in metastasis susceptibility. SUN2 is an integral membrane protein that spans the inner nuclear membrane and interacts with Nesprins embedded in the outer nuclear membrane [48,49]. The cytoplasmic tails of Nesprins interact with the cellular cytoskeleton, providing mechanical linkage between the plasma membrane and the nucleus [50]. In addition to positioning the nucleus within the cell, it is thought that the plasma-to-nuclear membrane linkage enables direct communication between extracellular environment and nucleus and is faster and more sensitive than typical biochemical signaling [51]. Extracellular microenvironment has become increasingly recognized as a critical factor in metastatic progression. A direct role of SIPA1, RRP1B and BRD4-SF in a metastatic cells ability to sense and respond to the surrounding extracellular matrix and cellular environment would provide additional insights as to why cells differentially activate the metastatic cascade depending on the composition of the surrounding stroma [52]. 

Third, these data suggest that BRD4-SF is not a direct competitive inhibitor of BRD4-LF since the histone binding and nuclear localization patterns differ between the two isoforms. BRD4-LF is part of the transcriptional elongation machinery and it binds the positive transcription elongation factor b (P-TEFb) to phosphorylate the C-terminus of RNA polymerase II to release the polymerase complex after mRNA capping [13,53,54]. BRD4-SF, despite binding acetylated chromatin, is localized to the transcriptionally inactive perinuclear region and lacks one of the P-TEFb binding domains [55]. This suggests that BRD4-SF may bind to facultative heterochromatin at the nuclear membrane that is poised for release from the perinuclear compartment and transcriptional activation upon mechanical signaling. This model is consistent with BRD4-SF binding RRP1B, which we previously demonstrated was a facultative heterochromatin-associated protein [15]. Alternatively, the two BRD4 isoforms may be associated with maintenance of differentiation. Recent studies have demonstrated that like nucleolar-associated chromatin domains, lamina-associated domains are A/T rich and consist of both constitutive and cell type specific (facultative) elements [56]. Facultative lamina-associated domains are predominantly nuclear membrane-associated in embryonic stem cells consistent with this being the default state in undifferentiated cells [56]. Thus, BRD4-LF, by remaining on DNA during mitosis, not only acts as a transcriptional memory tag but also actively positions cell type specific transcriptional domains in the nuclear matrix. In contrast, BRD4-SF would suppress differentiated transcriptional programs while enhancing embryonic pathways. This interpretation is consistent with the undifferentiated mesenchymal-like phenotype observed in BRD4-SF, BRD4-ΔC mutant [3] and BRD4-NUT midline carcinoma patients [57,58]. This is also consistent with the I-BET151 results, where inhibition of BRD4 would potentially induce epithelial-to-mesenchymal dedifferentiation thought to be essential for metastatic progression by removing the long isoform from chromatin, resulting in a reversion to a default undifferentiated nuclear architecture. 

In summary, we demonstrated that bromodomain inhibitors, while efficacious against primary tumors in a mouse model of aggressive breast cancer, are not effective anti-metastatic agents. To better understand how these agents might be used to reduce the morbidity and mortality of cancer, which is primarily due to metastatic disease, we characterized the pro-metastatic isoform of BRD4. These studies demonstrated that BRD4-SF is associated with the inner nuclear membrane in association with other metastasis susceptibility proteins. The localizations and interactions raise intriguing, though still speculative, possibilities about mechanism of action through mechanotransduction and/or differentiation. Additional work is ongoing to answer these and other questions raised by the current study.

## Supporting Information

Figure S1 **Mass spectrometry analysis of BRD4-SF.** (A) Modular structure of BRD4 isoforms. The two BRD4 isoforms share the same N-terminal domain except for the final three amino acids. BRD4-SF lacks the C-terminal domain of BRD4. (B) Schematic of the steps of immunoprecipitation/mass spectrometry protein-protein interaction analysis of HEK293 cells transiently transfected with FLAG- tagged BRD4-SF expression vector or control.
(TIF)

Figure S2 **BRD4-SF interacts with factors involved in a wide range of biological processes.** Gene ontological analysis of the biological processes of BRD4-SF mass spectrometry data using the Gene Ontology tool ToppGene Suite.
(TIF)

Figure S3 **Lamond lab cellular fractionation protocol.** Schematic representation of the detailed steps of the Lamond lab subcellular fractionation protocol.
(TIF)

Figure S4 **Gardner lab cellular fractionation protocol.** Schematic representation of the detailed steps of the second subcellular fractionation protocol obtained from Dr. Kevin Gardner’s laboratory.
(TIF)

Figure S5 **Myc-tagged BRD4-SF stably expressing HeLa cells express low levels of BRD4-SF.** Western blot analysis of HeLa cells stably expressing myc-tagged BRD4-SF or an empty vector using anti-myc antibody. Anti-β-actin antibody was used as a loading control.
(TIF)

Table S1 (DOC)

Table S2 (DOC)
