# Supplementary figures and images for: BRD4 Short Isoform Interacts with RRP1B, SIPA1 and Components of the LINC Complex at the Inner Face of the Nuclear Membrane
Source: PLoS One. 2013 Nov 19;8(11):e80746. doi: 10.1371/journal.pone.0080746 (PMC3834312; doi:10.1371/journal.pone.0080746)

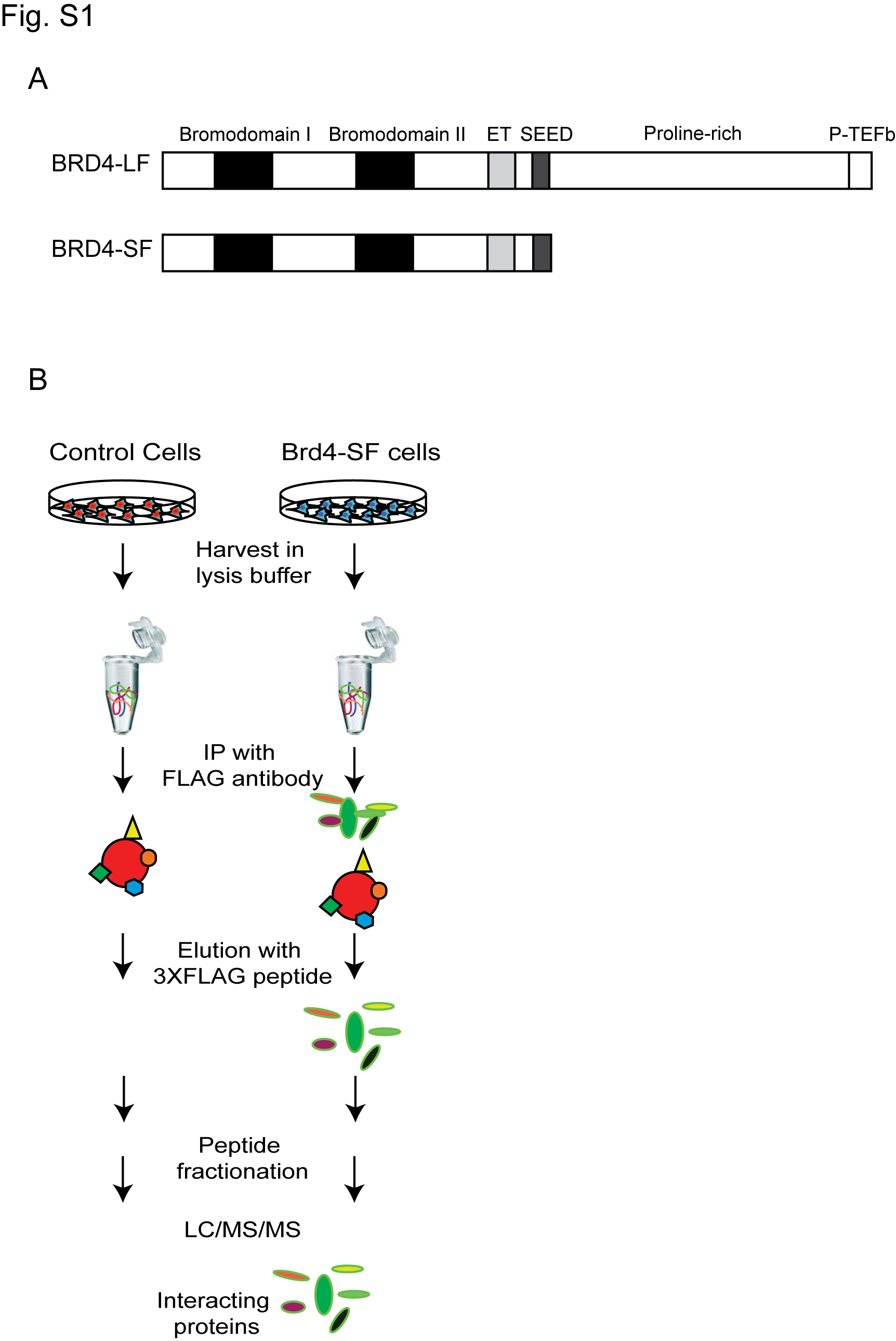

Supplement: Figure S1 — Mass spectrometry analysis of BRD4-SF. (A) Modular structure of BRD4 isoforms. The two BRD4 isoforms share the same N-terminal domain except for the final three amino acids. BRD4-SF lacks the C-terminal domain of BRD4. (B) Schematic of the steps of immunoprecipitation/mass spectrometry protein-protein interaction analysis of HEK293 cells transiently transfected with FLAG- tagged BRD4-SF expression vector or control. (TIF) [file pone.0080746.s001.tif]

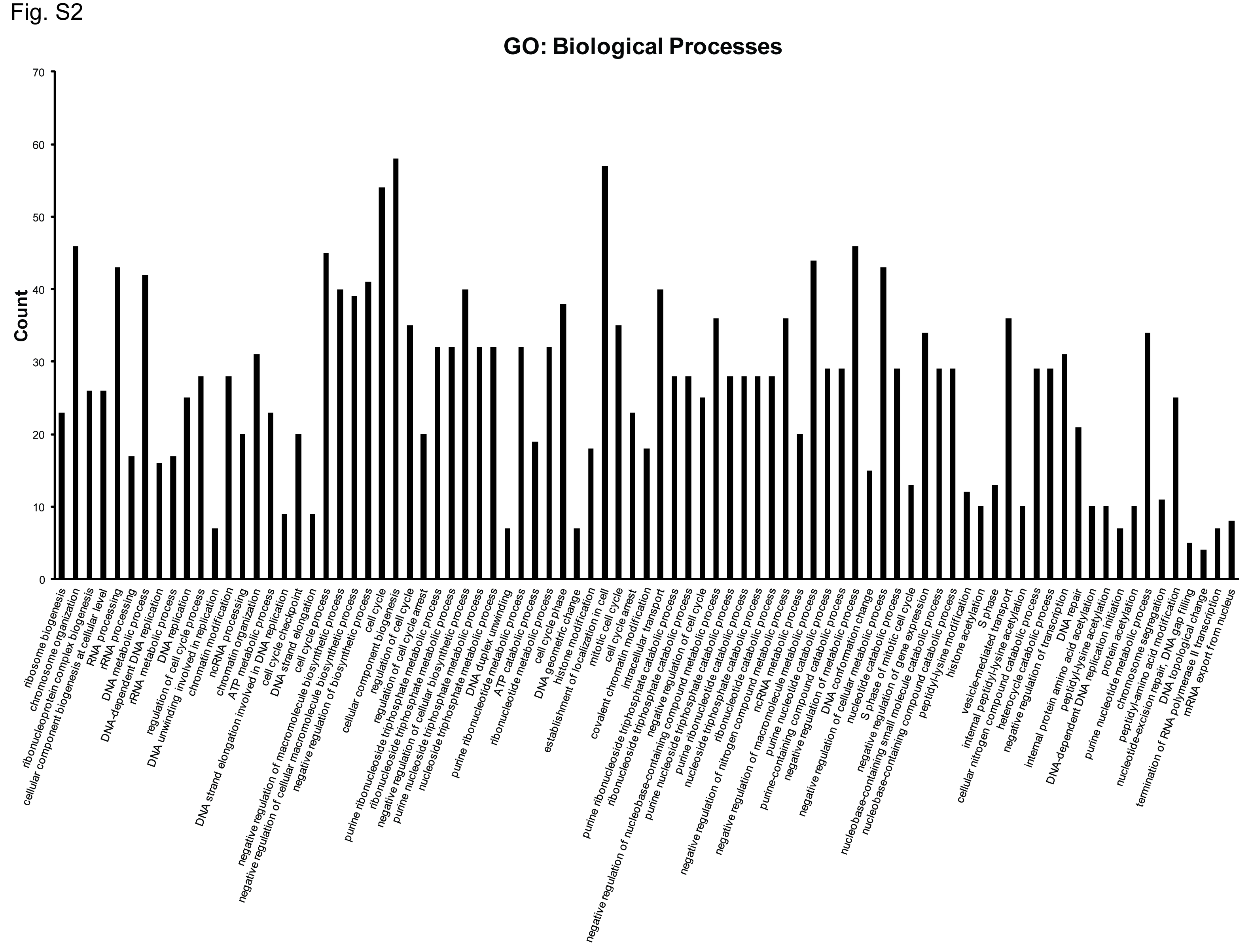

Supplement: Figure S2 — BRD4-SF interacts with factors involved in a wide range of biological processes. Gene ontological analysis of the biological processes of BRD4-SF mass spectrometry data using the Gene Ontology tool ToppGene Suite. (TIF) [file pone.0080746.s002.tif]

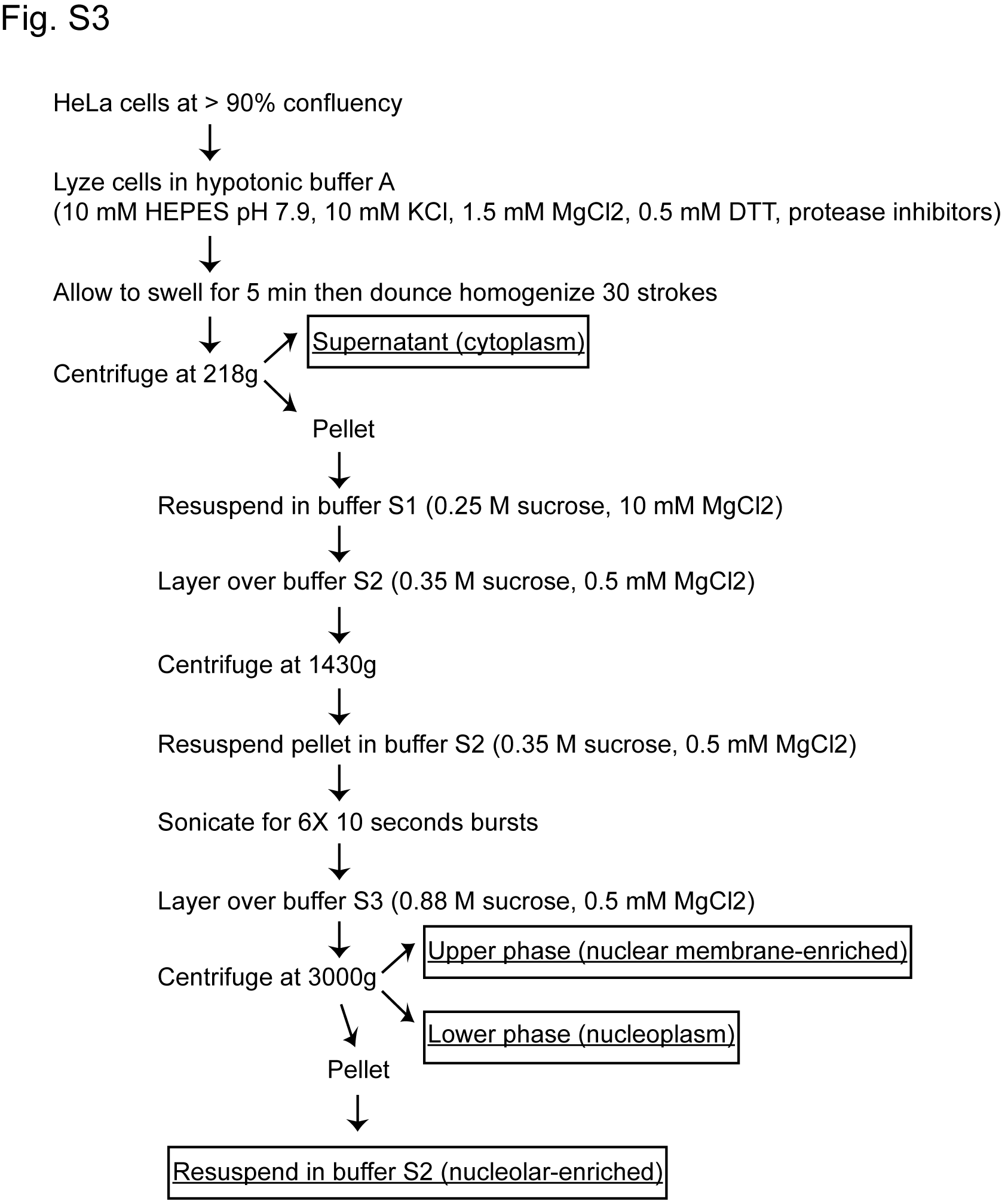

Supplement: Figure S3 — Lamond lab cellular fractionation protocol. Schematic representation of the detailed steps of the Lamond lab subcellular fractionation protocol. (TIF) [file pone.0080746.s003.tif]

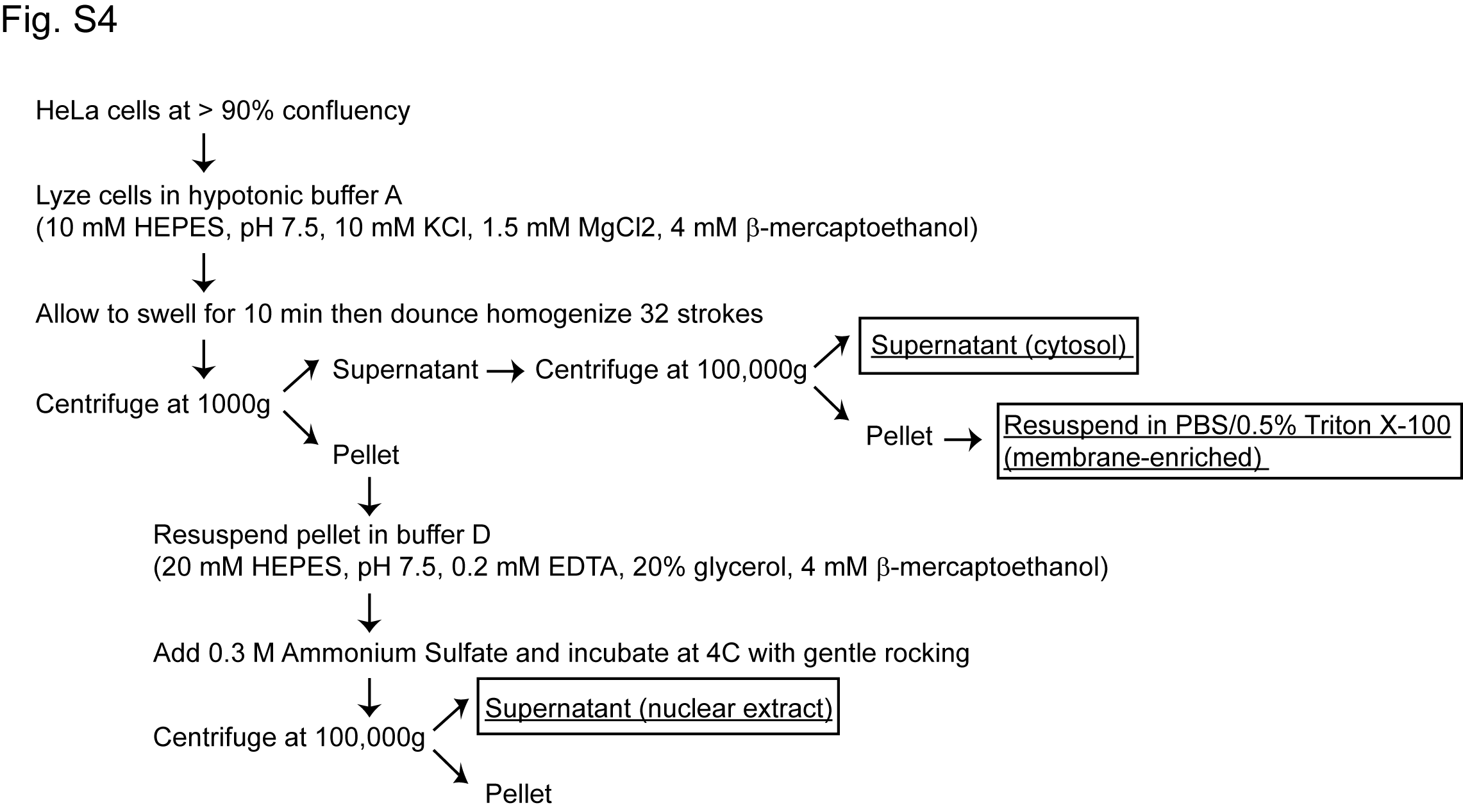

Supplement: Figure S4 — Gardner lab cellular fractionation protocol. Schematic representation of the detailed steps of the second subcellular fractionation protocol obtained from Dr. Kevin Gardner’s laboratory. (TIF) [file pone.0080746.s004.tif]

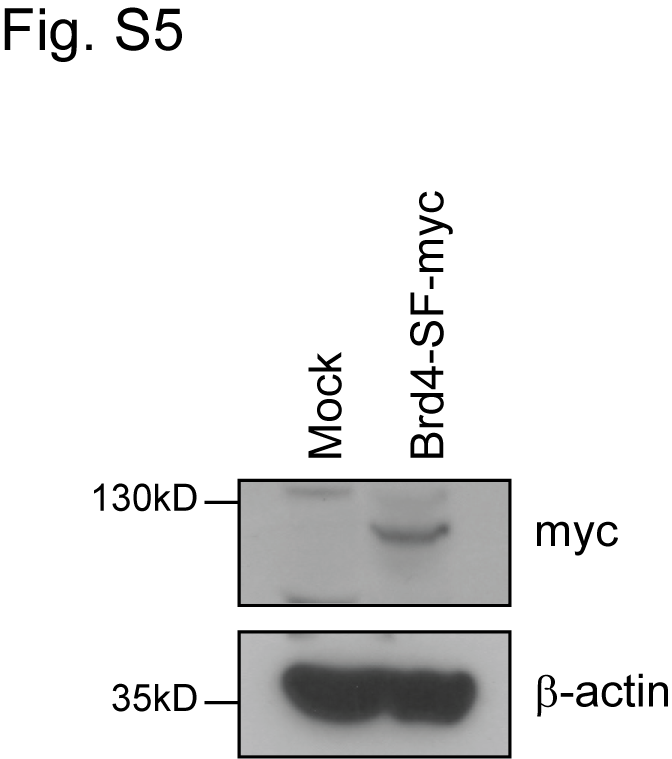

Supplement: Figure S5 — Myc-tagged BRD4-SF stably expressing HeLa cells express low levels of BRD4-SF. Western blot analysis of HeLa cells stably expressing myc-tagged BRD4-SF or an empty vector using anti-myc antibody. Anti-β-actin antibody was used as a loading control. (TIF) [file pone.0080746.s005.tif]
