# Supplementary material for: BRD4 Short Isoform Interacts with RRP1B, SIPA1 and Components of the LINC Complex at the Inner Face of the Nuclear Membrane
Source: PLoS One. 2013 Nov 19;8(11):e80746. doi: 10.1371/journal.pone.0080746 (PMC3834312; doi:10.1371/journal.pone.0080746)
Supplement: Table S1 — (DOC) [file pone.0080746.s006.doc]

| **Table S1. Nucleolar and Nuclear Envelope Proteins Interacting with BRD4-SF by MS** | | | | |
| --- | --- | --- | --- | --- |
| **Gene Symbol** | **Gene Name** | **Number of Interactions Detected With**  **BRD4-SF Control** | | |
| **Nucleolar proteins** | | | |  |
| **RRP1B** | Ribosomal RNA processing protein 1 homolog B | | 9 | 0 |
| **LYAR** | Cell growth-regulating nucleolar protein | | 7 | 0 |
| **NOG1** | Nucleolar GTP-binding protein 1 | | 6 | 0 |
| **NOP14** | Nucleolar protein 14 | | 5 | 0 |
| **PRP4** | U4/U6 small nuclear ribonucleoprotein Prp4 | | 5 | 0 |
| **NOC4L** | Nucleolar complex protein 4 homolog | | 4 | 0 |
| **NOG2** | Nucleolar GTP-binding protein 2 | | 4 | 0 |
| **NOL6** | Nucleolar protein 6 | | 4 | 0 |
| **NPA1P** | Nucleolar pre-ribosomal-associated protein 1 | | 4 | 0 |
| **UT14A** | U3 small nucleolar RNA-associated protein 14 homolog A | | 3 | 0 |
| **HNRL2** | Heterogeneous nuclear ribonucleoprotein U-like protein 2 | | 2 | 0 |
| **NO40** | Nucleolar protein of 40 kDa | | 2 | 0 |
| **NOL10** | Nucleolar protein 10 | | 2 | 0 |
| **NOL8** | Nucleolar protein 8 | | 2 | 0 |
| **PRPF3** | U4/U6 small nuclear ribonucleoprotein Prp3 | | 2 | 0 |
| **ROAA** | Heterogeneous nuclear ribonucleoprotein A/B | | 2 | 1 |
| **IMP3** | U3 small nucleolar ribonucleoprotein protein IMP3 | | 1 | 0 |
| **MK67I** | MKI67 FHA domain-interacting nucleolar phosphoprotein | | 1 | 0 |
| **NOC3L** | Nucleolar complex protein 3 homolog | | 1 | 0 |
| **NOL12** | Nucleolar protein 12 | | 1 | 0 |
| **NOP16** | Nucleolar protein 16 | | 1 | 0 |
| **NUSAP** | Nucleolar and spindle-associated protein 1 | | 1 | 0 |
| **RU17** | U1 small nuclear ribonucleoprotein 70 kDa | | 1 | 0 |
| **RU2A** | U2 small nuclear ribonucleoprotein A | | 1 | 0 |
| **RUXF** | Small nuclear ribonucleoprotein F | | 1 | 1 |
| **TDIF2** | Deoxynucleotidyltransferase terminal-interacting protein 2 | | 1 | 0 |
| **UBF1** | Nucleolar transcription factor 1 | | 1 | 0 |
| **UTP11** | Probable U3 small nucleolar RNA-associated protein 11 | | 1 | 0 |
| **Nuclear envelope-associated proteins** | | | |  |
| **JMJD6** | Bifunctional arginine demethylase and lysyl-hydroxylase JMJD6 | | 16 | 0 |
| **PARP1** | Poly [ADP-ribose] polymerase 1 | | 12 | 0 |
| **NAT10** | N-acetyltransferase 10 | | 11 | 0 |
| **TCOF** | Treacle protein | | 11 | 0 |
| **RRP1B** | Ribosomal RNA processing protein 1 homolog B | | 9 | 0 |
| **CEBPZ** | CCAAT/enhancer-binding protein zeta | | 8 | 0 |
| **SSRP1** | FACT complex subunit SSRP1 | | 8 | 1 |
| **DDX18** | ATP-dependent RNA helicase DDX18 | | 7 | 0 |
| **DHX30** | Putative ATP-dependent RNA helicase DHX30 | | 7 | 0 |
| **LYAR** | Cell growth-regulating nucleolar protein | | 7 | 0 |
| **SRPK1** | Serine/threonine-protein kinase SRPK1 | | 7 | 0 |
| **DDX24** | ATP-dependent RNA helicase DDX24 | | 6 | 0 |
| **TOP1** | DNA topoisomerase 1 | | 6 | 0 |
| **DDX51** | ATP-dependent RNA helicase DDX51 | | 5 | 0 |
| **DDX56** | Probable ATP-dependent RNA helicase DDX56 | | 5 | 0 |
| **GNL3** | Guanine nucleotide-binding protein-like 3 | | 5 | 0 |
| **PELP1** | Proline-, glutamic acid- and leucine-rich protein 1 | | 5 | 0 |
| **BMS1** | Ribosome biogenesis protein BMS1 homolog | | 4 | 0 |
| **CHERP** | Calcium homeostasis endoplasmic reticulum protein | | 4 | 0 |
| **DDX27** | Probable ATP-dependent RNA helicase DDX27 | | 4 | 0 |
| **DDX50** | ATP-dependent RNA helicase DDX50 | | 4 | 0 |
| **DDX54** | ATP-dependent RNA helicase DDX54 | | 4 | 0 |
| **MAN1** | Inner nuclear membrane protein Man1 | | 4 | 0 |
| **MCM2** | DNA replication licensing factor MCM2 | | 4 | 0 |
| **MCM7** | DNA replication licensing factor MCM7 | | 4 | 0 |
| **NOC4L** | Nucleolar complex protein 4 homolog | | 4 | 0 |
| **NOL6** | Nucleolar protein 6 | | 4 | 0 |
| **RAB7A** | Ras-related protein Rab-7a | | 4 | 0 |
| **SENP3** | Sentrin-specific protease 3 | | 4 | 0 |
| **TBL3** | Transducin beta-like protein 3 | | 4 | 0 |
| **WDR33** | WD repeat-containing protein 33 | | 4 | 0 |
| **WDR36** | WD repeat-containing protein 36 | | 4 | 0 |
| **CTCF** | Transcriptional repressor CTCF | | 3 | 0 |
| **LAS1L** | Protein LAS1 homolog | | 3 | 0 |
| **POP1** | Ribonucleases P/MRP protein subunit POP1 | | 3 | 0 |
| **RBM27** | RNA-binding protein 27 | | 3 | 0 |
| **RFC3** | Replication factor C subunit 3 | | 3 | 0 |
| **RRAS** | Ras-related protein R-Ras | | 3 | 1 |
| **RRP1** | Ribosomal RNA processing protein 1 homolog A | | 3 | 0 |
| **TEX10** | Testis-expressed sequence 10 protein | | 3 | 0 |
| **AATF** | Protein AATF | | 2 | 0 |
| **DDB1** | DNA damage-binding protein 1 | | 2 | 0 |
| **DDX10** | Probable ATP-dependent RNA helicase DDX10 | | 2 | 0 |
| **DDX47** | Probable ATP-dependent RNA helicase DDX47 | | 2 | 0 |
| **FXR1** | Fragile X mental retardation syndrome-related protein 1 | | 2 | 0 |
| **FXR2** | Fragile X mental retardation syndrome-related protein 2 | | 2 | 0 |
| **MCM4** | DNA replication licensing factor MCM4 | | 2 | 0 |
| **MCM6** | DNA replication licensing factor MCM6 | | 2 | 0 |
| **MDC1** | Mediator of DNA damage checkpoint protein 1 | | 2 | 0 |
| **NOL10** | Nucleolar protein 10 | | 2 | 0 |
| **PHC2** | Polyhomeotic-like protein 2 | | 2 | 0 |
| **PRPF3** | U4/U6 small nuclear ribonucleoprotein Prp3 | | 2 | 0 |
| **RNPS1** | RNA-binding protein with serine-rich domain 1 | | 2 | 0 |
| **RRP12** | RRP12-like protein | | 2 | 0 |
| **RRS1** | Ribosome biogenesis regulatory protein homolog | | 2 | 0 |
| **SMC3** | Structural maintenance of chromosomes protein 3 | | 2 | 0 |
| **SRP68** | Signal recognition particle 68 kDa protein | | 2 | 0 |
| **STT3B** | Dolichyl-diphosphooligosaccharide--protein glycosyltransferase subunit STT3B | | 2 | 0 |
| **TBL2** | Transducin beta-like protein 2 | | 2 | 0 |
| **TOP2A** | DNA topoisomerase 2-alpha | | 2 | 0 |
| **TSR1** | Pre-rRNA-processing protein TSR1 homolog | | 2 | 0 |
| **U2AF2** | Splicing factor U2AF 65 kDa subunit | | 2 | 0 |
| **UTP18** | U3 small nucleolar RNA-associated protein 18 homolog | | 2 | 0 |
| **WDR3** | WD repeat-containing protein 3 | | 2 | 0 |
| **ABT1** | Activator of basal transcription 1 | | 1 | 0 |
| **ACSL3** | Long-chain-fatty-acid-CoA ligase 3 | | 1 | 0 |
| **AP1G2** | AP-1 complex subunit gamma-like 2 | | 1 | 0 |
| **AP2A1** | AP-2 complex subunit alpha-1 | | 1 | 0 |
| **ARRB2** | Beta-arrestin-2 | | 1 | 0 |
| **ASPH** | Aspartyl/asparaginyl beta-hydroxylase | | 1 | 0 |
| **ATP5L** | ATP synthase subunit g, mitochondrial | | 1 | 0 |
| **BLK** | Tyrosine-protein kinase Blk | | 1 | 0 |
| **CD2AP** | CD2-associated protein | | 1 | 0 |
| **CNOT1** | CCR4-NOT transcription complex subunit 1 | | 1 | 0 |
| **COPG** | Coatomer subunit gamma | | 1 | 0 |
| **CPSF1** | Cleavage and polyadenylation specificity factor subunit 1 | | 1 | 0 |
| **CPSF3** | Cleavage and polyadenylation specificity factor subunit 3 | | 1 | 0 |
| **DPM1** | Dolichol-phosphate mannosyltransferase | | 1 | 0 |
| **DPY30** | Protein dpy-30 homolog | | 1 | 0 |
| **DRG1** | Developmentally-regulated GTP-binding protein 1 | | 1 | 0 |
| **DYSF** | Dysferlin | | 1 | 0 |
| **EIF3D** | Eukaryotic translation initiation factor 3 subunit D | | 1 | 0 |
| **ELF1** | ETS-related transcription factor Elf-1 | | 1 | 0 |
| **FRG1** | Protein FRG1 | | 1 | 0 |
| **GMFB** | Glia maturation factor beta | | 1 | 0 |
| **GSTP1** | Glutathione S-transferase P | | 1 | 0 |
| **HAX1** | HCLS1-associated protein X-1 | | 1 | 0 |
| **HDAC2** | Histone deacetylase 2 | | 1 | 0 |
| **LCP2** | Lymphocyte cytosolic protein 2 | | 1 | 0 |
| **LIMS1** | LIM and senescent cell antigen-like-containing domain protein 1 | | 1 | 0 |
| **MTA2** | Metastasis-associated protein MTA2 | | 1 | 0 |
| **MTX1** | Metaxin-1 | | 1 | 0 |
| **MYH14** | Myosin-14 | | 1 | 0 |
| **NOC3L** | Nucleolar complex protein 3 homolog | | 1 | 0 |
| **NUMA1** | Nuclear mitotic apparatus protein 1 | | 1 | 0 |
| **PDIA4** | Protein disulfide-isomerase A4 | | 1 | 0 |
| **PGM2** | Phosphoglucomutase-2 | | 1 | 0 |
| **PIGT** | GPI transamidase component PIG-T | | 1 | 0 |
| **PRDX1** | Peroxiredoxin-1 | | 1 | 0 |
| **PRDX6** | Peroxiredoxin-6 | | 1 | 0 |
| **PSPC1** | Paraspeckle component 1 | | 1 | 0 |
| **PURA** | Transcriptional activator protein Pur-alpha | | 1 | 0 |
| **PWP2** | Periodic tryptophan protein 2 homolog | | 1 | 0 |
| **RAB14** | Ras-related protein Rab-14 | | 1 | 0 |
| **RAB2A** | Ras-related protein Rab-2A | | 1 | 0 |
| **RAB6A** | Ras-related protein Rab-6A | | 1 | 0 |
| **RASA2** | Ras GTPase-activating protein 2 | | 1 | 0 |
| **RBM19** | Probable RNA-binding protein 19 | | 1 | 0 |
| **RBM23** | Probable RNA-binding protein 23 | | 1 | 0 |
| **RBM42** | RNA-binding protein 42 | | 1 | 0 |
| **RFC2** | Replication factor C subunit 2 | | 1 | 0 |
| **RPA2** | DNA-directed RNA polymerase I subunit RPA2 | | 1 | 0 |
| **RRBP1** | Ribosome-binding protein 1 | | 1 | 0 |
| **RTN3** | Reticulon-3 | | 1 | 1 |
| **SAR1A** | GTP-binding protein SAR1a | | 1 | 0 |
| **SCFD1** | Sec1 family domain-containing protein 1 | | 1 | 0 |
| **SIRT3** | NAD-dependent deacetylase sirtuin-3, mitochondrial | | 1 | 0 |
| **SPCS3** | Signal peptidase complex subunit 3 | | 1 | 0 |
| **SRPRB** | Signal recognition particle receptor subunit beta | | 1 | 0 |
| **TMED9** | Transmembrane emp24 domain-containing protein 9 | | 1 | 0 |
| **TOP2B** | DNA topoisomerase 2-beta | | 1 | 0 |
| **TRRAP** | Transformation/transcription domain-associated protein | | 1 | 0 |
| **UCHL5** | Ubiquitin carboxyl-terminal hydrolase isozyme L5 | | 1 | 0 |
| **WDR18** | WD repeat-containing protein 18 | | 1 | 0 |
| **WIZ** | Protein Wiz | | 1 | 0 |
| **XPO5** | Exportin-5 | | 1 | 0 |
| **XRCC1** | DNA repair protein XRCC1 | | 1 | 0 |
| **YLPM1** | YLP motif-containing protein 1 | | 1 | 0 |
| **ZAP70** | Tyrosine-protein kinase ZAP-70 | | 1 | 0 |
| **ZC3H4** | Zinc finger CCCH domain-containing protein 4 | | 1 | 0 |
| **ZFR** | Zinc finger RNA-binding protein | | 1 | 0 |
