# Supplementary material for: BRD4 Short Isoform Interacts with RRP1B, SIPA1 and Components of the LINC Complex at the Inner Face of the Nuclear Membrane
Source: PLoS One. 2013 Nov 19;8(11):e80746. doi: 10.1371/journal.pone.0080746 (PMC3834312; doi:10.1371/journal.pone.0080746)
Supplement: Table S2 — (DOC) [file pone.0080746.s007.doc]

| **Table S2. Proteins Interacting with Both BRD4-SF and RRP1B** | | | | | |
| --- | --- | --- | --- | --- | --- |
| **Gene symbol** | **Gene full name** | **Number of interactions detected with** | | | |
|  |  | **BRD4-SF** | **Control** | **RRP1B** | **Control** |
| **RPL10A** | 60S ribosomal protein L10a | 13 | 0 | 3 | 0 |
| **PARP1** | Poly (ADP-ribose) polymerase 1 | 12 | 0 | 2 | 0 |
| **RRP1B** | Ribosomal RNA processing protein 1 homolog B | 9 | 0 | 15 | 0 |
| **TOP1** | DNA topoisomerase 1 | 6 | 0 | 2 | 0 |
| **PRPF4B** | Serine/threonine-protein kinase PRP4 homolog | 5 | 0 | 1 | 0 |
| **RSL1D1** | Ribosomal L1 domain-containing protein 1 | 3 | 0 | 2 | 0 |
| **PPP1CA** | Serine/threonine-protein phosphatase PP1-alpha catalytic subunit | 1 | 0 | 2 | 0 |
